# Supplementary figures and images for: Chemical-mineralogical features and physical properties of archaeological adobe: The evidence from Tell Zurghul/Nigin (Dhi Qar, Iraq)
Source: PLoS One. 2026 Feb 23;21(2):e0342009. doi: 10.1371/journal.pone.0342009 (PMC12928471; doi:10.1371/journal.pone.0342009)

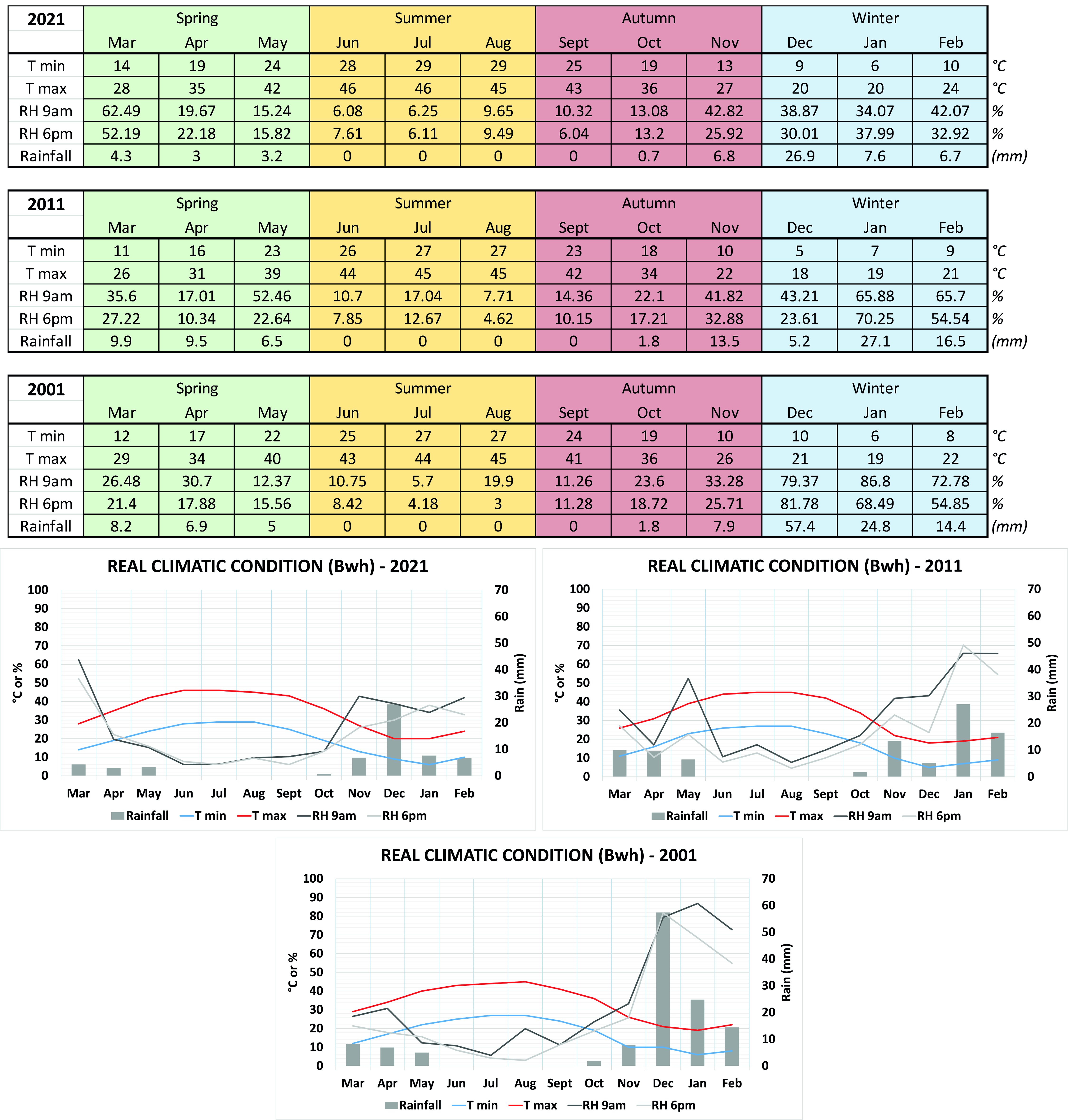

Supplement: S1 Fig — Supplementary information in relation to climatic data for the Tell Zurghul/Nigin area from the WorldClim database at https://worldclim.org/. (TIF) [file pone.0342009.s001.tif]

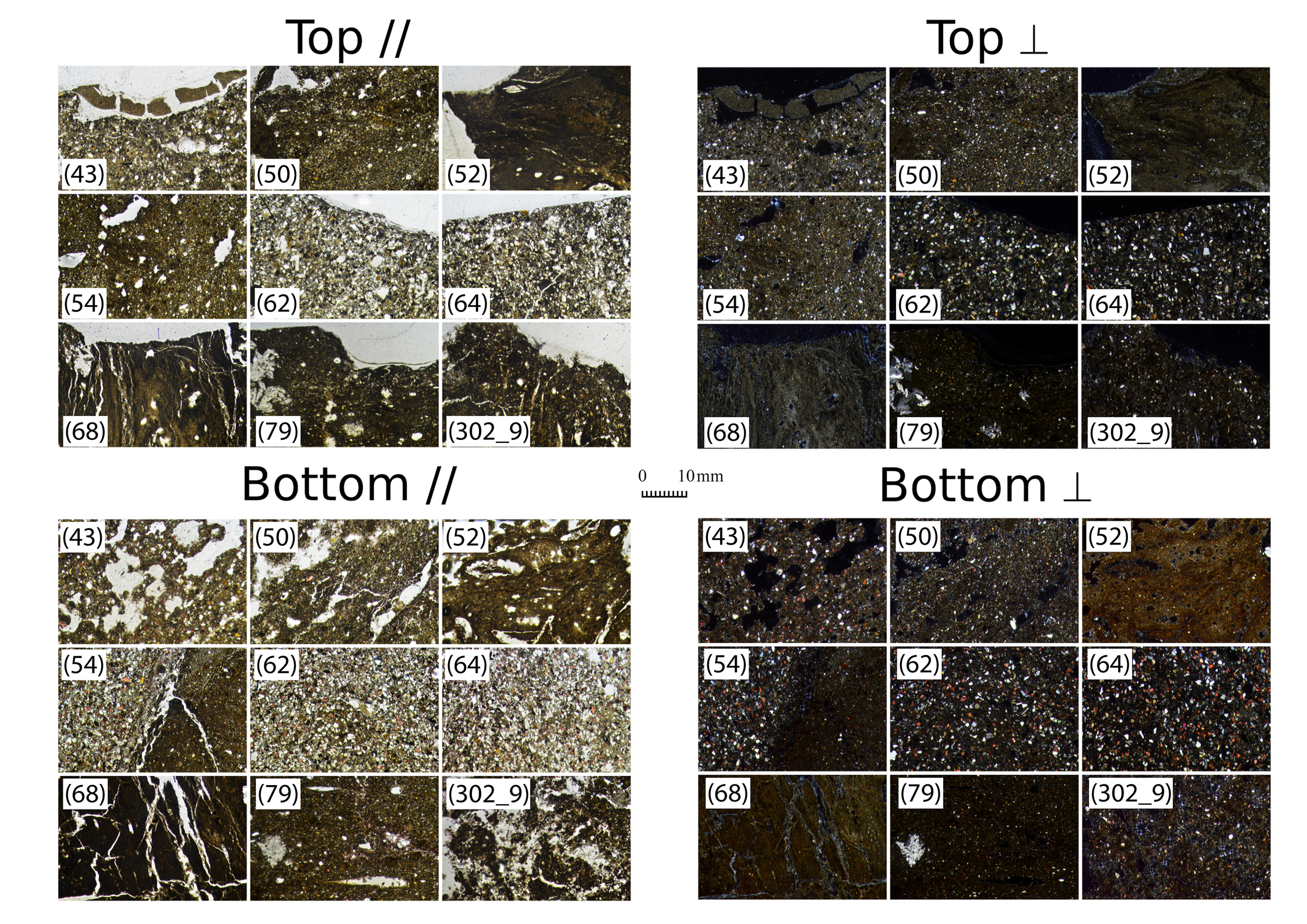

Supplement: S2 Fig — (TIF) [file pone.0342009.s002.tif]
