# Supplementary material for: Chemical-mineralogical features and physical properties of archaeological adobe: The evidence from Tell Zurghul/Nigin (Dhi Qar, Iraq)
Source: PLoS One. 2026 Feb 23;21(2):e0342009. doi: 10.1371/journal.pone.0342009 (PMC12928471; doi:10.1371/journal.pone.0342009)
Supplement: Attachment — Submitted filename: PONE-D-25-34039_reviewer.pdf [file pone.0342009.s007.pdf]

PLOS ONE

Chemical-mineralogical features and physical properties of archaeological mudbricks. The evidence from Tell Zurghul/Nigin (Dhi Qar, Iraq) in view of conservative solutions

--Manuscript Draft--

**Manuscript Number:**	PONE-D-25-34039
**Article Type:**	Research Article
**Full Title:**	Chemical-mineralogical features and physical properties of archaeological mudbricks. The evidence from Tell Zurghul/Nigin (Dhi Qar, Iraq) in view of conservative solutions
**Short Title:**	Chemical-mineralogical and physical properties of archaeological mudbricks
**Corresponding Author:**	Luca Volpi, Ph.D.<br>Autonomous University of Madrid: Universidad Autonoma de Madrid<br>Madrid, SPAIN
**Keywords:**	Mesopotamia; Earthen architecture; Mineralogy; Petrography; Durability; Conservation
**Abstract:**	This study examines the chemical, mineralogical, hydric and mechanical properties of archaeological earthen mudbricks and earthen plasters from the site of Tell Zurghul/Nigin in southern Iraq, which are associated with recently excavated buildings dated to the 5th and 3rd millennium BCE.<br>As integral components of earthen architecture, mudbrick structures are susceptible to rapid deterioration when subjected to environmental conditions and other degradation factors. Consequently, their preservation is a critical focus within cultural heritage initiatives involving earthen constructions. A comprehensive understanding of the materials used in buildings and their inherent properties is essential for identifying optimal conservation methods for archaeological earthen structures.<br>The research is driven by two primary objectives. Firstly, it seeks to explore construction techniques within a diachronic framework, assessing their temporal evolution while considering the geological context and availability of local raw materials. Secondly, mineralogical and chemical analyses, alongside hydric and mechanical tests, aim to evaluate the characteristics of mudbricks, including their resistance and durability against weathering. The results are intended to inform future conservation strategies both at the local and broader regional levels.
**Order of Authors:**	Luca Volpi
	Francesco Santoro De Vico
	Anna Arizzi
	Nicola Lanzaro
	Davide Nadali
**Additional Information:**	
**Question**	**Response**
**Financial Disclosure**<br><br>Enter a financial disclosure statement that describes the sources of funding for the work included in this submission. Review the submission guidelines for detailed requirements. View published research articles from *PLOS ONE* for specific examples.<br><br>This statement is required for submission	Yes

and **will appear in the published article** if the submission is accepted. Please make sure it is accurate.<br><br>**Funded studies**<br>Enter a statement with the following details:<br>• Initials of the authors who received each award<br>• Grant numbers awarded to each author<br>• The full name of each funder<br>• URL of each funder website<br>• Did the sponsors or funders play any role in the study design, data collection and analysis, decision to publish, or preparation of the manuscript?<br><br>Did you receive funding for this work?	
Please add funding details.<br>as follow-up to "**Financial Disclosure**<br><br>Enter a financial disclosure statement that describes the sources of funding for the work included in this submission. Review the submission guidelines for detailed requirements. View published research articles from *PLOS ONE* for specific examples.<br><br>This statement is required for submission and **will appear in the published article** if the submission is accepted. Please make sure it is accurate.<br><br>**Funded studies**<br>Enter a statement with the following details:<br>• Initials of the authors who received each award<br>• Grant numbers awarded to each author<br>• The full name of each funder<br>• URL of each funder website<br>• Did the sponsors or funders play any role in the study design, data collection and analysis, decision to publish, or preparation of the manuscript?<br><br>Did you receive funding for this work?"	The CIVIS3i-MSCA research project EnEAp – Endangered Earthen Architecture project. New Challenges in the Conservation and Restoration Practices of Archaeological Earthen Masonries in Western Asia (2022-EnEAp-231; PI: Luca Volpi) has received funding from the European Union's Horizon 2020 research and innovation programme under grant agreement N°101034324.<br>This research was also funded by the MICIU/AEI/10.13039/501100011033 and FEDER, UE, under the project PID2023-146405OB-100 (2024-2027).

Please select the country of your main research funder (please select carefully as in some cases this is used in fee calculation).<br>as follow-up to "**Financial Disclosure**<br><br>Enter a financial disclosure statement that describes the sources of funding for the work included in this submission. Review the submission guidelines for detailed requirements. View published research articles from *PLOS ONE* for specific examples.<br><br>This statement is required for submission and **will appear in the published article** if the submission is accepted. Please make sure it is accurate.<br><br>**Funded studies**<br>Enter a statement with the following details:<br>• Initials of the authors who received each award<br>• Grant numbers awarded to each author<br>• The full name of each funder<br>• URL of each funder website<br>• Did the sponsors or funders play any role in the study design, data collection and analysis, decision to publish, or preparation of the manuscript?<br><br>Did you receive funding for this work?"	SPAIN - ES
**Competing Interests**<br><br>Use the instructions below to enter a competing interest statement for this submission. On behalf of all authors, disclose any competing interests that could be perceived to bias this work—acknowledging all financial support and any other relevant financial or non-financial competing interests.<br><br>This statement is required for submission and **will appear in the published article** if the submission is accepted. Please make sure it is accurate and that any funding	The authors have declared that no competing interests exist.

sources listed in your Funding Information later in the submission form are also declared in your Financial Disclosure statement.<br><br>View published research articles from *PLOS ONE* for specific examples.<br><br>**NO authors have competing interests**<br><br>Enter: *The authors have declared that no competing interests exist.*<br><br>**Authors with competing interests**<br><br>Enter competing interest details beginning with this statement:<br><br>*I have read the journal's policy and the authors of this manuscript have the following competing interests: [insert competing interests here]*<br><br>* typeset	
**Ethics Statement**<br><br>Enter an ethics statement for this submission. This statement is required if the study involved:<br><br>• Human participants<br>• Human specimens or tissue<br>• Vertebrate animals or cephalopods<br>• Vertebrate embryos or tissues<br>• Field research<br><br>Write "N/A" if the submission does not require an ethics statement.<br><br>General guidance is provided below. Consult the submission guidelines for detailed instructions. **Make sure that all information entered here is included in the Methods section of the manuscript.**	N/A

**Format for specific study types**<br><br>**Human Subject Research (involving human participants and/or tissue)**<br>• Give the name of the institutional review board or ethics committee that approved the study<br>• Include the approval number and/or a statement indicating approval of this research<br>• Indicate the form of consent obtained (written/oral) or the reason that consent was not obtained (e.g. the data were analyzed anonymously)<br><br>**Animal Research (involving vertebrate animals, embryos or tissues)**<br>• Provide the name of the Institutional Animal Care and Use Committee (IACUC) or other relevant ethics board that reviewed the study protocol, and indicate whether they approved this research or granted a formal waiver of ethical approval<br>• Include an approval number if one was obtained<br>• If the study involved *non-human primates*, add *additional details* about animal welfare and steps taken to ameliorate suffering<br>• If anesthesia, euthanasia, or any kind of animal sacrifice is part of the study, include briefly which substances and/or methods were applied<br><br>**Field Research**<br><br>Include the following details if this study involves the collection of plant, animal, or other materials from a natural setting:<br>• Field permit number<br>• Name of the institution or relevant body that granted permission	
**Data Availability**<br><br>Authors are required to make all data underlying the findings described fully available, without restriction, and from the time of publication. PLOS allows rare exceptions to address legal and ethical concerns. See the PLOS Data Policy and FAQ for detailed information.	Yes - all data are fully available without restriction

A Data Availability Statement describing where the data can be found is required at submission. Your answers to this question constitute the Data Availability Statement and **will be published in the article**, if accepted.<br><br>**Important:** Stating 'data available on request from the author' is not sufficient. If your data are only available upon request, select 'No' for the first question and explain your exceptional situation in the text box.<br><br>Do the authors confirm that all data underlying the findings described in their manuscript are fully available without restriction?	
**Describe where the data may be found in full sentences. If you are copying our sample text, replace any instances of XXX with the appropriate details.**<br><br>• If the data are **held or will be held in a public repository**, include URLs, accession numbers or DOIs. If this information will only be available after acceptance, indicate this by ticking the box below. For example: *All XXX files are available from the XXX database (accession number(s) XXX, XXX.).*<br>• If the data are all contained **within the manuscript and/or Supporting Information files**, enter the following: *All relevant data are within the manuscript and its Supporting Information files.*<br>• If neither of these applies but you are able to provide **details of access elsewhere**, with or without limitations, please do so. For example:<br><br>*Data cannot be shared publicly because of [XXX]. Data are available from the XXX Institutional Data Access / Ethics Committee (contact via XXX) for researchers who meet the criteria for access to confidential data.*<br><br>*The data underlying the results presented in the study are available from (include the name of the third party*	All data are available under CC-BY-SA-4.0 license from the e-cienciaDatos Online Repository (https://doi.org/10.21950/Z7EFXL)

and contact information or URL).<br>• This text is appropriate if the data are owned by a third party and authors do not have permission to share the data.<br><br>* typeset	
Additional data availability information:	Tick here if the URLs/accession numbers/DOIs will be available only after acceptance of the manuscript for publication so that we can ensure their inclusion before publication.

Powered by Editorial Manager® and ProduXion Manager® from Aries Systems Corporation

Manuscript Click here to access/download;Manuscript;Volpi et al. Mudbrick Zurghul_PlosOne_manuscript.docx

Chemical-mineralogical features and physical properties of archaeological mudbricks. The evidence from Tell Zurghul/Nigin (Dhi Qar, Iraq) in view of conservative solutions

Luca Volpi[1] *, Francesco Santoro De Vico[2], Anna Arizzi[3], Nicola Lanzaro[4], Davide Nadali[4]

[1] Departamento de Historia Antigua, Historia Medieval y Paleografía y Diplomática, Universidad Autónoma de Madrid, Madrid, Spain

[2] "Giuseppe Colombo" University Center for Space Studies and Activities (CISAS), University of Padova, Padova, Italy

[3] Departamento de Mineralogía y Petrología, Facultad de Ciencias, Universidad de Granada, Granada, Spain

[4] Dipartimento di Scienze dell'Antichità, Sapienza Università di Roma, Roma, Italy

*luca.volpi@uam.es

Abstract

This study examines the chemical, mineralogical, hydric and mechanical properties of archaeological earthen mudbricks and earthen plasters from the site of Tell Zurghul/Nigin in southern Iraq, which are associated with recently excavated buildings dated to the 5th and 3rd millennium BCE.

As integral components of earthen architecture, mudbrick structures are susceptible to rapid deterioration when subjected to environmental conditions and other degradation factors. Consequently, their preservation is a critical focus within cultural heritage initiatives involving earthen constructions. A comprehensive understanding of the materials used in buildings and their inherent properties is essential for identifying optimal conservation methods for archaeological earthen structures.

The research is driven by two primary objectives. Firstly, it seeks to explore construction techniques within a diachronic framework, assessing their temporal evolution while considering the geological context and availability of local raw materials. Secondly, mineralogical and chemical analyses, alongside hydric and mechanical tests, aim to evaluate the characteristics of mudbricks, including their resistance and durability against weathering. The results are intended to inform future conservation strategies both at the local and broader regional levels.

Keywords

Mesopotamia, Earthen architecture, Mineralogy, Petrography, Durability, Conservation

1. Introduction

Once excavated, archaeological earthen masonries are quickly re-exposed to the decay agents, as they were during their period of use [1]. However, whereas in the past earthen structures were originally surface-plastered and protected from precipitation by the roof, after modern excavation, earthen constructions are directly exposed to natural elements without further protection [2].

In this paper, we investigate mudbrick architecture, considered as part of a larger set of building techniques collectively referred to as earthen architecture (including cobb, rammed earth – tapial or pisé –, and wattle and daub; [3-5]). The selected case-study area is West Asia, a region in which the use of earthen masonries is substantial due to the presence of important river-basins (i.e., the Euphrates and the Tigris rivers) which participates in creating an environment rich in mud and clay, and due to the climatic conditions of the area. Its widespread presence and cost-effectiveness have made clay one of the main building materials in the area from prehistoric times to the present day [6].

When dealing with the excavation of mudbrick architecture, several questions arise as to the appropriateness of adopting integrated solutions for their conservation (see, e.g., [7, 8]), and current

procedures for protecting archaeological earthen structures are mainly based on four approaches: 1) backfilling [1: 79-85, 9-12]; 2) the construction of temporary or permanent protective shelters [1: 117-120, 13-22]; 3) limited interventions on the structures using newly-made locally produced mudbricks (e.g., for encapsulation and/or capping, or by filling voids and cracks with mortar, and by plastering the mudbricks with a lime-clay-straw mixture; [1: 86-91, 23-30]; for the complete reconstruction of a mudbrick massive structure, see [31]); 4) interventions on the structures using stabilising materials as a hardener (in particular, ethyl-silicate and aqueous and acrylic polymeric solutions have been utilised; [1: 92-97, 32-37]).

The detailed understanding of building materials and their chemical-physical properties and mechanical resistance is as relevant as the identification of the best preservation technique to be used on archaeological earthen structures [38-45]. The present research intends to investigate the properties of unfired bricks and plasters used for building purposes during the 5th and the 3rd millennium BCE at the archaeological site of Tell Zurghul/Nigin (Nasiriyah) in southern Iraq [46-50].

Two main objectives are recognised: on the one hand, the interest to investigate construction techniques at both a synchronic and diachronic level in an important site of ancient Mesopotamia, together with their consistency with the geological context of the area and the local raw materials. On the other hand, the study of their chemical-mineralogical features was carried out, and hydric and mechanical tests were run in order to determine the main characteristics of the mudbricks, along with their resistance and durability against weathering, as an insight for future conservation strategies at the site (local scale) and in the area (macroscale).

2. Archaeological, Geological and Climatic Context

2.1 Archaeological setting

Tell Zurghul/Nigin (31°22'36.06"N; 46°29'36.24"E) is an archaeological site located in south-eastern Iraq (Dhi Qar). The site is approximately 40 km north-east of Nasiriyah, the capital city of the Dhi Qar Governorate, and ca. 7 km south-east of the ancient Sumerian city of Lagash, present-day Tell al-Hiba. Since 2014, an Italian archaeological expedition of Sapienza University of Rome (MAIN: Missione Archeologica Italiana a Tell Zurghul/Nigin) is regularly working with yearly seasons of excavation. The settlement covers a surface of about 70 hectares, and it has been uninterruptedly occupied from the mid-5th to the end of the 3rd millennium BCE, when the city was destroyed and never extensively re-occupied. The site was part of the ancient State of Lagash, and, thanks to several cuneiform inscriptions, it has been identified with the Sumerian city of Nigin. The site landscape is characterised by two extensive mounds, called Mound A and Mound B (Fig. 1). The first one corresponds to the main occupational phases of the ancient city of Nigin already known by cuneiform sources (3rd millennium BCE; [46-48]). Mound B is located some meters south-west from Mound A, and it is mainly occupied in the prehistoric phases of the site (mid-5th millennium BCE; [49, 50]).

Fig. 1 Topographic map of the Tell Zurghul/Nigin site with the indication of surveyed and excavated areas (© Missione Archeologica Italiana a Nigin). Mound A has been investigated in several operations (Area A, D, and E); Mound B has been investigated in one operation named Area B.

2.2 Geology of the area and climatic conditions

The Nasiriyah region, situated within the Lower Mesopotamian Plain (LMP) of southern Iraq's Dhi Qar Governorate, represents a critical transition zone between the fluvio-deltaic systems of the Tigris-Euphrates rivers and the Persian Gulf [51]. Geomorphologically, the area is characterised by Quaternary alluvial sediments overlying an unstable shelf zone created by the collision of the Arabian plate with the Zagros Fold-and-Thrust Belt [52, 53] (Fig. 2).

Fig. 2 Geological map of Iraq (after [53]).

This tectonic setting has resulted in a subsiding basin with a northwest-southeast trending fault system that fundamentally influences hydrological patterns and sedimentation processes. The Nasiriyah area exhibits a complex stratigraphy that reflects multiple depositional environments, including fluvial, lacustrine, and episodic marine influences. Sedimentological evidence, including the Hammar Formation, indicates that marine transgression reached this region during the mid-Holocene [54]. The contemporary landscape manifests a low-gradient topography (approximately 3-5m above MSL) with distinctive anastomosing river patterns as the Euphrates approaches the Hammar Lake zone. This geomorphological configuration has played a crucial role in shaping human settlement patterns since the Ubaid period, with the Nasiriyah area functioning as an interstitial ecological zone where fluvial deposition, climate fluctuations, and human adaptations have interacted throughout the Holocene [55].

Tell Zurghul (Nasiriyah, Iraq) is the selected site spot, characterised by a semi-arid (BWh) climate as classified by Köppen-Geiger (https://koeppen-geiger.vu-wien.ac.at/; [56]).

Southern Iraq features a hot desert climate with extremely hot summers, where temperatures regularly exceed 40°C, particularly in July and August. The region receives minimal annual rainfall, less than 100 mm, mostly occurring between November and April. Rainfall regime is torrential (Fig. 3).

Fig. 3 Tell Zurghul/Nigin real climatic conditions, mean values from years 2021-2011-2001 (data from the WorldClim database at https://worldclim.org/; S1 File for further information).

3. Materials and methods

3.1. Provenience of archaeological samples

Thirty archaeological samples (29 mudbricks and one earthen plaster sample) were collected during the 2021–2022 excavation seasons at Tell Zurghul/Nigin by MAIN, and exported with the permission of the SBAH (State Board of Antiquities and Heritage) in Baghdad (Tab. 1).

Tab. 1 ID samples, provenience, archaeological phasing and dating.

ID	Wall N.	Arch. Ph.	Arch. Period	Type
43	W.389	Mound B, Step V	Uruk	Brick
44	W.292	\	Late Ubaid	Brick
45	W.386	Mound B, AP 5	Late Ubaid	Brick
46	W.389	Mound B, Step V	Uruk	Brick
47	W.381	Mound B, AP 4	Late Ubaid	Brick
48	SU 861	Mound B, AP 6	Late Ubaid	Brick
49	W.389	Mound B, Step V	Uruk	Brick
50	SU 861	Mound B, AP 6	Late Ubaid	Brick
51	W.382	Mound B, AP 4	Late Ubaid	Brick
52	W.382	Mound B, AP 4	Late Ubaid	Brick
53	W.389	Mound B, Step V	Uruk	Brick
54	W.386	Mound B, AP 5	Late Ubaid	Brick
55	W.381	Mound B, AP 4	Late Ubaid	Brick
56	W.361	Mound B, AP 2c	Late Ubaid	Brick
57	W.381	Mound B, AP 4	Late Ubaid	Brick
58	SU 861	Mound B, AP 6	Late Ubaid	Brick
59	W.386	Mound B, AP 5	Late Ubaid	Brick
60	W.382	Mound B, AP 4	Late Ubaid	Brick
61	W.362	Mound B, AP 2b	Late Ubaid	Brick
62	W.264	Mound B, AP 2b	Late Ubaid	Brick
63	W.362	Mound B, AP 2b	Late Ubaid	Brick
64	W.701	Mound B, AP 3a	Late Ubaid	Brick
65	\	\	Late Ubaid	Brick
66	W.701	Mound B, AP 3a	Late Ubaid	Brick
67	W.361	Mound B, AP 2c	Late Ubaid	Brick
68	W.361	Mound B, AP 2c	Late Ubaid	Brick
69	W.701	Mound B, AP 3a	Late Ubaid	Brick
79	W.458	Mound A	Mid-3rd Mill. BCE	Brick
80	W.458	Mound A	Mid-3rd Mill. BCE	Brick
SG.22.E.302/9	W.458	Mound A	Mid-3rd Mill. BCE	Plaster

The selection criteria are based on the desire to investigate construction techniques at a synchronic level and their evolution over time. Therefore, three to four samples for each architectural phase in Mound B and three samples from Mound A have been selected. Only a single sample of earthen plaster has been

selected, as it is the sole well-preserved specimen identified to date at Tell Zurghul/Nigin, and it was included primarily to compare its composition and use of local clay sources with the mudbricks samples. Twenty-seven mudbrick samples come from Mound B, and they are dated to the mid-5th millennium BCE. Archaeologically, associated pottery materials are assigned to the Late Ubaid (Ubaid 4) phase. The operations focused on two different portions of Mound B: an extensive area (ca. 20 x 16 m) has been investigated on the top of the mound since 2015 and aimed at the comprehension of the overall surface architectural layouts and their stratigraphic sequence; the second area is a step trench (ca. 30 x 3 m) excavated in 2021 in the northern slope of the mound to investigate the earliest occupation of the mound [49, 50]. Nine mudbrick samples from the operation on top of the mound come from wall structures belonging to different stratigraphic layer and architectural phases (henceforth, AP) 2b, AP 2c, and AP 3a (numerical order is from most recent to earliest; Fig. 4A). Twelve mudbrick samples come from the step trench, belonging to AP 4, AP 5, and AP 6. At the base of the step trench, an archaeological layer dated to the mid-4th millennium BCE (Step V) have been found. Four mudbrick samples originate from walls associated with this level (Fig. 4B). Two mudbrick samples come from the excavations in Mound B, but they are not associated with any architectural phase.

Fig. 4 A) Area B: reconstructed section of the excavation at top of the mound (APs 1–3); B) Area B: reconstructed section of Step Trench excavation in 2021 (APs 4–6); c) Area E: plan of the 2022 excavation season.

Two samples of mudbricks and one earthen plaster come from Mound A, and they are dated to the mid-3rd millennium BCE. Samples come from Area E, a 15 x 20 m trench, located on the south-western slope of Mound A. The operations of the 2019 and 2022 seasons aimed to detect the occupational nature and sequence of the mound, investigating the morphology of the hill in connection with the northernmost Area D (excavated in 2016 and 2019; [46, 48] and the southernmost Area A at the base of the mound

(excavated in 2015, 2016, and 2017; [57, 58]). They belong to a wall structure (W.458) dated to the ED IIIB/early Akkadian horizon by pottery (Fig. 4C).

3.2 Archaeological and autoptic group distinction

Samples are divided into groups based on their archaeological provenience and their visual distinction. This subdivision was done prior to any archaeometric analysis in order to group the samples according to visual features, and based on the following parameters: compactness, texture, and colour at the naked eye. Six groups have been identified (Fig. 5).

Fig. 5 Mudbrick samples (photos) for each Autoptic Group (1–6).

Group 1 is characterised by a compact matrix with few visible voids, and a buff-light beige colour. Group 2 is distinguished from the former by a darker brown colour. Samples in group 3 have a granular appearance with clay arranged in small lumps, and with a dark beige-greyish colour. Group 4 is very compact, with a more clayey appearance and a light beige colour. Group 5 shows a higher appearance of sand and organic inclusions (shell). Group 6 is composed of all 3rd millennium BCE samples (both mudbricks and earthen plaster) with an orange colour (Tab. 2).

Tab. 2 Distribution of samples across autoptic groups.

	Group 1	*Group 2*	*Group 3*	*Group 4*	*Group 5*	*Group 6*
**ID**	43, 44, 45, 46, 47, 48, 50, 54, 55,	49, 53, 56, 67	51, 52, 68	61, 62, 63	64, 65	79, 80 302/9

	57, 58, 59, 60, 66, 69					

3.3 Characterisation and testing methods

3.3.1 Chemical, mineralogical, and petrographic study

5g of each sample was powdered in an agate mill in order to perform chemical and mineralogical analysis (30 samples).

Chemical analysis was carried out by X-ray fluorescence (XRF) with an XRF energy disperser spectrometer PANalytical Zetium, which integrates a tube with a rhodium anode, an X ray generator of 4 kW, a decoupled goniometer θ/2θ, and proportional detectors of scintillation and high-level flow. The analysis was performed on all samples and was used to determine and quantify major and trace elements in the samples' composition [59: 331-399].

Mineralogical analysis was performed by Powder X-ray diffraction (PXRD) using a X'Pert Pro Diffractometer equipped with continuous sample rotation system using the disordered crystalline powder method. The experimental conditions were as follows: voltage of 45 kV; current of 40 mA; CuKα radiation (λ= 1.5405 Å); explored area between 4° and 70° 2θ; goniometer speed of 0.1° 2θ/s. The results were analysed with the X'Pert Highscore Plus 4.9 software (PANalytical), equipped with the Joint Committee for Powder Diffraction Standards (JCPDS) database. The analysis was performed on all 30 samples and was used to determine their crystalline structures [59: 430-438].

Polished mudbrick thin-sections of 9 (out of 30) samples were analysed under a polarized optical microscopy Carl Zeiss "Jenapol-U" equipped with a Nikon D7000 reflex camera. The selection is based on a multitude of factors, which include, among others: the size of the original sample to obtain a thin section that would allow sufficient material to be preserved for further tests; the attempt to cover, as broadly as possible, the spectrum of macroscopic differences recognised in the differentiation into

autoptic groups; the inclusion of two samples dating to the 3rd millennium BCE (as opposed to the others dating to the 5th millennium BCE). Mudbricks were stabilized with epoxy resin before being cut for realising polished thin sections. The petrographic characterisation was used to determine texture, granulometry, porosity, nature of clay and mineral components, and presence/absence of organic materials [59: 21-45].

3.3.2 Colour

A spectrophotometer CM-700d Konica Minolta, with a 3 mm diameter (SAV) and a CM-S100W DATA Software COLOR SpectraMagic NX was used for the colorimetry test. Colour analysis was performed on the original samples (30 samples), without further preparations.

Colour properties of the mudbricks were registered following the EN 15886:2010 standard. The obtained data is expressed using the chromatic parameters of the CIELAB system: L* (luminosity), a* (green to red spectrum), b* (blue to yellow spectrum), h* (tone/shade), and C* (chroma).

3.3.3 Compactness and strength

Compactness and strength tests (Scotch Tape Test, and Surface Hardness Test) were performed on the original samples (30 samples), without further preparations.

The Scotch Tape Test (or Peeling Test) was performed to assess the surface cohesion of the samples, and to provide an indication of the surface deterioration of the mudbricks.

The detached material from each sample was analysed through visual inspection (photos) and weight measurement, following [60]. The weighting was conducted based on ASTM D3359 regulation, with a ±0,0001 g precision scale. 2.6 x 3.1 paper labels (ca. 8 cm^2) with double-sided tape were prepared and weighted (unladen weight). The paper labels were attached to a flat sample face (both surface and core) with homogenous pressure (up to six pressures, adhesion time: 0.30 min). The labels were stripped off

(tear-off angle of ca. 90°) and re-weighed to quantify the amount of material remaining adhered on the tape. Two to three measurements were taken on each sample, depending on their nature and preservation. The Surface Hardness test was performed with a portable hardness tester with a striker PCE-2500N (PCE Ibérica). Measurements were taken on the real mudbrick surface, when available (26 samples; among the other four, two are too small in size to apply the measurement, two have values below the instrument's minimum threshold). Ten measurements were taken for each sample in order to have representative values and an average.

3.3.4 Hydric and rain resistance tests

Surfaces and horizontal fresh cut sherds of mudbricks were prepared for static contact angle and imbibition test (30 samples).

The static contact angle (CA) of mudbrick samples was measured by the sessile drop method (4 µL of MilliQ® water) on a Data Physics OCA 15EC device equipped with Peltier stage (25 °C) and SCA20 (Version 5.0.41) software (see [61]). Two measurements for each sample were performed, one on the real mudbrick surface (R), and one on a fresh-cut fracture of the mudbrick core (F).

The imbibition time test of both real and fresh-cut surfaces has been measured through video analysis.

Both static contact angle (CA) and imbibition time test have also been performed to 5 cubes with a side of ca. 2 cm used for the rain resistance test (see below). Tests have been performed on two surfaces for each cube.

Rain resistance tests are carried out to evaluate the impact of environmental conditions on various materials (i.e., mudbricks) in an accelerated time (see, e.g., [62]). The test enables the replication of specific climatic conditions within a controlled laboratory environment, allowing the decay process to be parameterised over a defined time period. The analysis was performed to simulate the environmental conditions (in particular rainfall) to which mudbrick structures are exposed in Southern Iraq.

The temperature ramp was applied during each wetting period, with the test conducted in precision oven "Digitronic-TFT", SELECTA®, maintaining controlled temperature ranges (from room temperature to an average of the maximum temperatures recorded in the area in years 2001-2011-2021 as depicted in Fig. 3, with the aim of exposing the samples to maximum thermal stress for each cycle).

One year of rainfall variations was simulated over four days, with each season represented by a one-day test thus simulating a one-year stress. Before each daily cycle, the corresponding seasonal rainfall was applied using a pipette. Water application methods include immersion, drop-by-drop with a pipette, or using a spray, each representing different rainfall intensities. Considering the rainfall characteristics in Southern Iraq, derived from differential precipitation regimes and intensity-duration-frequency (IDF) curves [63], Nasiriyah experiences torrential rain. In order to simulate torrential rain, pouring all the water at once is preferable. We used a 3 mL pipette, with each drop weighing approximately 50 mg (0.05 mL), to apply the entire quantity of water in a short period of time in order to simulate torrential rain (S2 File). Relative humidity was not set as a fixed value, as it varied as a function of the temperature tested in each period/season.

Pre-wetting and wetting conditions were monitored using weight measurements, photogrammetry, and calibre measurements. Data were registered at the start of each daily cycle.

Climatic data were sourced from the WorldClim database (https://worldclim.org/) and plotted using QGIS to collect climatic data on specific spots (30-second image resolution).

Rainfall (mm; Fig. 3) per month was recorded for three reference years (2001, 2011, and 2021). Average monthly seasonal precipitations for the three reference years (aggregated for three months per season) were calculated for the simulation.

Cubes of ca. 2 cm per side were prepared for the rain resistance test (5 samples, nos. 50, 52, 55, 62, and 79). To calculate the amount of water to be applied, the rainfall in millimetres was converted into volume. Each millimetre of rainfall equals 1 litre per square meter (1 L/m²), or 1,000 millilitres per 10,000 square

centimetres (mL/10,000 cm²). Since 1 square meter (m²) = 10,000 square centimetres (cm²), the volume of water per square centimetre is 0.1 mL per square centimetre (mL/cm²). The final formula to calculate the water volume V (in mL) to apply to a surface A (cm²) is:

$$V(mL) = \frac{h(mm) \times A(cm^2)}{10}$$

where h is the rainfall depth in millimetres (mm) and A is the area in square centimetres (cm²).

4 Results

4.2 Chemical, mineralogical, and petrographic study

4.2.1 X-ray fluorescence (XRF)

XRF data indicate that the chemical composition of all analysed samples is relatively uniform (Tab. 3).

Tab. 3 Chemical composition of the 30 studied samples by means of X-Ray Fluorescence (XRF). The oxides content is given in (%).

Sample	SiO2	Al2O3	Fe2O3	MnO	MgO	CaO	Na2O	K2O	TiO2	P2O5	Zr	LOI
43	41.72	9.42	5.16	0.10	5.98	14.84	2.74	1.59	0.60	0.28	107.8	17.56
44	39.04	10.09	5.03	0.09	4.35	16.64	2.07	1.96	0.58	0.14	99.0	19.99
45	38.40	9.16	5.21	0.09	6.01	14.23	4.16	1.49	0.59	0.15	103.5	20.50
46	41.72	9.49	5.20	0.09	6.05	14.53	2.55	1.63	0.62	0.24	106.1	17.87
47	42.01	9.72	5.35	0.10	5.87	13.98	2.75	1.61	0.60	0.15	101.5	17.85
48	39.80	9.60	5.50	0.10	6.45	14.61	2.88	1.57	0.63	0.15	102.7	18.68
49	40.56	9.33	5.22	0.09	6.41	14.54	2.53	1.66	0.60	0.20	105.8	18.85
50	40.40	9.45	5.54	0.10	5.88	14.76	2.63	1.58	0.62	0.14	109.1	18.88
51	40.02	11.00	5.83	0.10	5.33	14.48	1.57	2.17	0.61	0.15	98.4	18.72
52	39.12	10.55	5.53	0.10	5.14	15.57	1.80	2.02	0.59	0.14	97.4	19.03
53	40.65	9.28	5.14	0.09	6.50	14.11	2.67	1.65	0.60	0.19	105.4	19.10
54	41.77	9.72	5.46	0.10	6.22	15.10	2.10	1.56	0.63	0.17	114.2	16.89

55	42.06	9.65	5.32	0.09	5.81	14.27	2.78	1.58	0.60	0.14	98.0	17.68
56	42.17	12.89	6.77	0.11	5.67	10.58	2.25	2.59	0.66	0.14	108.2	16.16
57	41.93	9.87	5.57	0.10	6.04	14.22	2.35	1.64	0.63	0.15	102.3	17.47
58	39.45	9.47	5.40	0.10	6.33	14.85	2.97	1.57	0.60	0.15	102.0	19.09
59	40.98	9.74	5.59	0.10	6.30	15.20	2.25	1.55	0.65	0.15	118.8	17.36
60	38.62	10.32	5.41	0.10	5.16	16.87	1.75	1.95	0.56	0.14	93.7	18.54
61	43.44	8.86	4.12	0.08	4.99	15.99	2.38	1.63	0.54	0.31	98.0	17.07
62	43.70	8.84	4.10	0.09	4.96	15.43	2.79	1.62	0.53	0.33	98.0	17.43
63	43.69	8.76	4.00	0.08	4.95	15.98	2.55	1.58	0.53	0.25	103.3	17.62
64	44.71	8.89	4.04	0.08	4.85	15.67	2.53	1.57	0.54	0.29	98.7	16.81
65	38.32	8.40	4.19	0.09	5.58	17.31	2.65	1.70	0.48	0.32	101.0	20.94
66	43.66	9.41	4.68	0.10	4.98	16.07	1.94	1.71	0.56	0.27	104.3	16.21
67	42.23	12.47	6.60	0.11	5.46	11.72	1.90	2.50	0.65	0.15	107.0	15.61
68	40.47	11.53	6.16	0.10	5.39	14.42	1.89	2.28	0.59	0.14	97.2	16.48
69	44.19	8.70	3.95	0.08	4.85	15.99	2.51	1.55	0.52	0.30	100.5	16.77
79	40.55	9.99	5.57	0.10	6.07	15.71	1.73	1.87	0.61	0.16	97.3	17.14
80	41.25	10.14	5.71	0.10	6.10	15.38	1.57	1.88	0.63	0.15	98.9	16.67
SG22E302/9	40.05	9.93	5.55	0.10	5.98	14.55	2.17	1.98	0.61	0.21	100.6	18.85

Variations are limited to a few percentage points and appear to result from random factors rather than deliberate ancient technical or cultural choices. Based on abundance criteria, the raw materials used seem to appear consistently the same and locally sourced.

Silicon (expressed as SiO_2 in Tab. 3) is among the most abundant elements, with variances between 38 and 44 %. It is mainly found in quartz, feldspars and other silicates, minerals present in significant quantities in all samples, as discussed in section 4.2.2.

Calcium oxide (CaO) mostly derives from calcite and dolomite minerals, both identified in the mineralogical analysis, while magnesium oxide (MgO) is predominantly found in minerals such as

dolomite and serpentine. Sodium oxide (Na_2O) is abundant in minerals of the feldspar group, particularly plagioclases.

The Principal Component Analysis (PCA) was carried out on the chemical data through Python 3.8+ (following the script indicated in S3). PCA shows that most samples (except samples nos. 56 and 67) falls within the 95.5 % variation, suggesting general uniformity in chemical composition (Fig. 6). Some sub-groupings can be identified: the largest group of samples shows a relationship between Na_2O and MgO variables; samples nos. 61, 62, 63, 64, and 69 (in the bottom left quadrant of the plot) show higher CaO content; some samples (in the bottom right quadrant of the graph), including samples nos. 56 and 67, show higher Al_2O_3 and K_2O content; the earthen plaster sample no. 302/9 is isolated from the other samples by its higher Fe_2O_3 content.

Fig. 6 Principal Component Analysis (PCA) carried out on the XRF data.

4.2.2 Powder X-ray diffraction (PXRD)

The mineralogical composition of the samples suggests the same consistency in raw material procurement and use, as already indicated by XRF analysis (Tab. 4).

Tab. 4 Mineralogical composition of the 30 studied samples obtained by qualitative Powder X-ray Diffraction (PXRD).

ID	Quartz	Calcite	Halite	Feldspars (s.l.) Plagioclases	Gypsum	Mica	Chlorite	Dolomite	Amphiboles	Serpentine	Sillimanite	Palygorskite	Orthopyroxene	Clinopyroxene	Rutile
**43**	X	X	X	X	X	X	X	X	X	X					
**44**	X	X	X	-	X	X	X	X							
**45**	X	X	X	X	X	X	X	X	X	X					
**46**	X	X		X	X	X	X	X						X	
**47**	X	X	X	X	X	X	X	X	X	X					
**48**	X	X	X	X	X	X	X	X	X						
**49**	X	X	X	X	X	X	X	X							
**50**	X	X	X	X		X	X	X		X					
**51**	X	X	X	X	X	X	X	X				X			
**52**	X	X	X	X	X	X	X	X							
**53**	X	X		X		X	X	X			X				
**54**	X	X	X	X		X	X	X		X		X			
**55**	X	X	X	X	X	X	X	X							
**56**	X	X	X	X	X		X					X			
**57**	X	X	X	X	X		X	X				X			
**58**	X	X	X	X	X	X	X	X							
**59**	X	X	X	X	X	X	X	X	X			X			
**60**	X	X	X	X	X	X	X	X							
**61**	X	X	X		X	X	X	X	X				X		
**62**	X	X			X	X	X	X	X			X			
**63**	X	X	X	X	X	X	X	X				X			

**64**	X	X	X	X	X	X	X	X	X	X			X		X
**65**	X	X	X	X	X	X	X			X			X		
**66**	X	X	X	X	X	X	X						X		
**67**	X	X	X	X	X	X	X								
**68**	X	X	X	X	X		X					X			
**69**	X	X		X	X	X	X		X	X	X		X		
**79**	X	X		X	X	X	X	X							
**80**	X	X	X	X	X	X	X	X							
**302/9**	X	X	X	X		X	X	X							

305

Quartz (SiO_2) and calcite ($CaCO_3$) are the predominant mineralogical phases in all samples (Fig. 7).

Fig. 7 X-ray Diffraction patterns of 10 samples with indication of the main mineral phases identified.

As the analysis performed was qualitative rather than quantitative, it is currently not possible to discern whether differences in quartz and calcite peaks correspond to actual differences in raw materials use or are due to random factors (such as the selection of one portion of the sample to be powdered instead of another, due to sample heterogeneity). The abundance of Ca, previously observed by XRF, is justified by the presence of dolomite ($CaMg(CO_3)_2$) in almost all samples.

The presence of halite (NaCl) can be explained by soil characteristics. In southern Mesopotamia, the soil is subject to continuous salinisation processes [64]. This phenomenon is documented in cuneiform texts for the 3rd and 2nd millennium BCE [65-69]. Therefore, the presence of halite may be attributed to post-depositional factors rather than its deliberate use in mudbrick production.

Plagioclase feldspars ($(Na, Ca)\ (Si, Al)_4O_8$) are present in almost every sample, predominantly in their calcium-rich mineralogical phase as anorthite ($CaAl_2Si_2O_8$).

Gypsum ($CaSO_4 \cdot 2H_2O$) is another mineral consistently found in almost all samples. It occurs naturally in southern Mesopotamia; its presence can therefore be explained by its local occurrence, while it remains to be determined whether it is present only residually or was deliberately added to the mudbricks production recipe.

Phyllosilicate minerals of the mica group (in particular, muscovite), and of the chlorite group (clinochlore) are also commonly attested in all analysed samples.

Some mineralogical phases are rarer and not present in all analysed samples (amphiboles, serpentine, sillimanite, palygorskite, orthopyroxene, clinopyroxene, and rutile). Notably, minerals from the

amphiboles group (hornblende, as seen in the XRD plots) are attested in 9 of 30 samples; interestingly, they appear in samples nos. 61, 62, 64, and 69, most of which form a sub-group in the chemical PCA.

4.2.3 Polarized optical microscopy (POM)

Petrographic analysis of thin sections from nine mudbrick samples revealed distinct textural and mineralogical characteristics (Fig. 10; S4 Figure).

Fig. 8 Selection of the polarised optical microscopy (POM) images obtained on three representative samples (nos. 62, 54, 68 respectively) under plane-polarized light (A, B, C) and cross-polarized light (D, E, F).

The primary mineral assemblage consists of quartz, calcite, feldspars, and dolomite as inclusions, while mica, chlorite, and serpentine constitute the binding phases. The samples exhibit varying porosity patterns, with some showing vertically oriented pores in the upper portions and more rounded, interconnected pores in the core. Several specimens display a clear structural differentiation between surface and core regions, with denser matrices observed near the surfaces.

Sample no. 54 exhibits a distinct division between a left zone containing abundant inert materials in the mud crack mixture and a right zone with a high percentage of clay binder.

Sample no. 62 stands out for its high aggregate content and minimal matrix, featuring predominantly crystalline inclusions with angular edges. Halite was identified in multiple samples, particularly on surfaces, possibly indicating weathering processes, as evidenced by its absence in the better-preserved sample no. 62. The matrix-to-aggregate ratio varies significantly across samples, with specimens like nos. 68 and 79 showing clay-rich, matrix-dominated compositions with sparse crystalline inclusions. Sample no. 68 also displays multiple fractures with two preferential fracture orientations. A notable feature in the earthen plaster sample no. 302/9 is the presence of extensive interconnected elongated

pores and fractures throughout the section, resembling root patterns. The porosity characteristics vary from compressed bi-dimensional pores with diagonal cross-orientation (sample no. 52) to more homogeneous circular voids (sample no. 54), reflecting different manufacturing techniques or post-depositional processes. Matrix composition and density show lateral variations within individual samples, particularly evident in sample no. 54, where the left side contains significantly more grains compared to the matrix-rich right side.

Tab. 5 Petrographic characteristics of mudbrick samples from archaeological context based on polarized optical microscopy analysis.

Sample	Primary Mineral Assemblage	Binding Phases	Matrix-Aggregate Ratio	Porosity Characteristics	Structural Features	Weathering/Special Notes
**43**	Calcite, quartz, feldspars, gypsum, dolomite, amphiboles	Serpentinite, mica, chlorite	Variable: matrix-enriched in central portion	Upper section: vertical pores. Core: rounded, interconnected pores	Oriented feldspars in core. Dense matrix at surface. Calcite and feldspar accumulation around pores	Halite present on surface. Large clay and mica inclusion
**50**	Calcite, feldspars, quartz, dolomite	Chlorite, mica, serpentinite	Matrix-rich	Surface: few horizontally oriented pores. Middle: large horizontal pores. Lower: small, rounded horizontal pores	Clay-enriched zones. Clay aggregates. Interconnected porosity in central zone	Presence of large clay nodules

**52**	Quartz, calcite, feldspars, gypsum, dolomite	Mica, chlorite	High clay content, minimal inert material	Upper section: compressed bi-dimensional pores with diagonal cross-orientation. Central section: dense with large voids. Core: small chaotic pores and large interconnected voids	Bipartite structure: left zone highly compact, right zone with large voids. Large clay aggregates	Structurally weak matrix. Clay aggregates without inclusions connected by fractures
**54**	Quartz, calcite, halite, feldspars, dolomite	Mica, chlorite, serpentinite, palygorskite	Variable: left section grain-rich, right section fine matrix-dominant	Relatively homogeneous, circular porosity. Lower section with smaller voids and denser structure	Distinct lateral division: left side with numerous grains, right side matrix-rich. Porosity along interface between zones	Halite present
**62**	Quartz, calcite, gypsum, mica, dolomite, amphiboles, palygorskite	Minimal chlorite	Very high aggregate content, minimal matrix	Large pores in upper section with east-west orientation. Very limited microporosity	Central fracture. Homogeneous inert material distribution. Angular crystalline inclusions	Absence of halite: potentially less weathered specimen. Angular crystals possibly intentionally crushed
**64**	Quartz, calcite, feldspars, gypsum, mica, dolomite, amphiboles, serpentinite, orthopyroxenes	Chlorite	Higher matrix content than sample 62, but still aggregate-rich	Predominantly circular pores. Few but extensive oblique northeast-southwest fractures	Relatively homogeneous structure. Highly crystalline	Localized clay-enriched zones. Potential rutile presence (requires SEM verification)
**68**	Quartz, calcite, halite, feldspars, gypsum	Chlorite, palygorskite	Very high matrix content, minimal aggregates	Limited, concentric porosity	Extensive horizontal and vertical fracturing. Laminated structure	Halite present

**79**	Quartz, calcite, feldspars, gypsum, mica, dolomite	Chlorite	High matrix content, limited inert material	Oriented voids	Larger plagioclase and quartz inclusions dispersed in matrix. Globular masses of feldspar and quartz. Plagioclase twinning	Mineral nodules present
**302/9**	Quartz, calcite, halite, feldspars, mica, dolomite	Chlorite	Relatively low aggregate to matrix ratio	Numerous elongated, interconnected pores. Few small pores. Multi-directional orientation	Extensive interconnected fractures throughout section. Quartz and feldspar nodules	Root-like pattern of interconnected elongated porosity. Halite present
**Note: All microphotographs were taken with north-south orientation.**						

4.3 Colour

Colour measurements are relevant for parametrising visual properties of archaeological materials to develop future conservation strategies.

The obtained results show that most samples share similar chromatic characteristics (S5 File). Lightness (L* parameter; Fig. 9A) is generally ranged between mean values of 50 and 65 %, with only two exceptions: sample no. 53 (mean value 47.27 %) and sample no. 51 (mean value 65.99 %).

Fig. 9 Chromatic parameters of the 30 analysed samples: A) L* (mean); B) a*-b* (mean).

The a* (green to red spectrum) and b* (blue to yellow spectrum) values are comprised between 3–7 a* (mean) and 13–20 b* (mean). Values are evenly distributed along a median line (Fig. 9B), with samples nos. 61, 62, 63, 64, 68, and 69 standing slightly apart in the lower left portion of the median line (having a* mean values comprised between 3–4, and b* mean values comprised between 13–16), in contrast to the other samples, which are distributed between 4.4–6.8 a* (mean), and 14.8–19.1 b* (mean).

4.4 Surface compactness and strength

Results of the Scotch Tape Test, measured in mg/cm^2, show varying degrees of material loss after adhesive tape application (Fig. 10A).

Fig. 10 A) Scotch Tape test plot; B) Surface Hardness test plot.

Samples exhibiting a material loss between 0–2 mg/cm^2 demonstrate the best surface cohesiveness, including samples nos. 44, 47, 49, 56, 59, 61, 63, and earthen plaster sample no. 302/9. On the contrary, samples showing more disaggregated surfaces and, therefore, major loss of adhered materials are nos. 46, and 55 (material loss > 7 mg/cm^2). The remaining samples exhibit material loss between 2–6 mg/cm^2. Surface cohesiveness can be correlated with Surface Hardness test data (Fig. 10B). In some cases, the preserved sample surfaces were insufficient to perform the test (samples nos. 44, and 47) or not hard enough to withstand the striker percussion (samples nos. 50, and 64, from which no results could be retrieved; S6 File). Mean values range from 181.8 HL (sample no. 79) and 300.9 HL (sample no. 51). Even after excluding 2 higher and 2 lower values, samples nos. 79 and 51 remain, respectively, the weakest (179.3 HL) and the strongest (286.3 HL). Samples nos. 43, 52, 55, and 67 are also very weak in terms of surface hardness. Without the two higher values, these samples present all values < 200 HL. Based on the mean of the six higher values (threshold 230 HL), samples nos. 45, 46, 49, 57, 59, 60, 63, 68, and 80 also present a strong surface hardness.

Correlating surface cohesiveness with surface hardness test data reveal that samples nos. 49, 59 and 63 gave the best results in both tests, having the highest hardness and the highest surface cohesion, while sample no. 55 is one with the lowest surface cohesion values and the lowest hardness.

4.5 Hydric and rain resistance tests

No static contact angle was identified for any of the 30 samples on either fresh section or actual surfaces. First contact angle was measured for only few samples on actual surfaces (samples nos. 43, 44, 46, 49, 53, 58, and 63). Regarding the imbibition time test, we generally observe that the imbibition time (s) is higher on actual surfaces than on fresh sections. Time values of both surfaces and fresh sections are generally below 1 s, indicating the samples are highly porous. In only two cases (samples nos. 44, and 46) is the imbibition time higher than 6 s (S7 File).

When the hydric test was performed on cubes of approximately 2 cm per side used for the rain resistance test (samples nos. 50, 52, 55, 62, and 79), which were previously saw-cut and sanded, similar results were obtained. Possibly due to sanding, the imbibition time is slightly higher than the first test (> 1 s) for all the samples, except for samples nos. 62_1, 62_2, and 79_2. The cube that shows highest imbibition values is sample no. 52, with a time comprised between 2.8 and 6.3 s. The sample that shows lowest imbibition values is sample no. 62, with a time comprised between 0.5 and 0.8 s.

The rain resistance test, conducted over four days and simulating one year rainfall variations, highlighted the critical weakness of mudbricks when exposed to decay agents, specifically rainfall. Test results (Fig. 11) show progressive material disaggregation coinciding with water application (steps 0, 2, and 3). Disaggregation leads to mass loss (Δg), which is directly proportional to the quantity (mL) of applied water, according to the different seasons reproduced in the laboratory.

Fig. 11 Rain resistance test, ΔM (mass), ΔV (volume) and Δρ (density) modification through cycles.

In terms of cycle duration, sample no. 79 shows clear signs of disaggregation and mass loss from the first water application, and already after the first cycle it loses almost half of its size and volume (ΔV), while it is the only sample that shows a significant expansion in density (Δρ) due to water absorption. After

cycle 2, the sample has already lost all its material cohesion, resulting in a formless mass of clay. Samples nos. 50, 52, and 55 show better results in terms of cohesiveness against weathering agents, with a progressive degradation after each water cycle. They show consistent ΔM, ΔV, and Δρ variations until step 3, while the winter water application has resulted in a great reduction of ΔM and ΔV, accompanied by a significant increase Δρ for sample no. 52. At the end of the test, samples nos. 50, 52, and 55 have lost all their cohesiveness. The mudbrick with the best water resistance is sample no. 62, which exhibits the most consistent Δ values, retaining its cubic shape while showing a slow but progressive loss of material with each water cycle. Moreover, during water application sample no. 62 is the only one to have shown minimal clay material loss.

Results have been standardised calculating volume and mass variations as if the cubes were precisely 2 cm per side. This was done to parametrise the data and to allow for comparisons (Fig. 12).

Fig. 12 Rain resistance test: result visualisation.

5 Discussion

Scientific analyses, combined with archaeological data, yielded significant results regarding sample composition and durability in decay tests.

From an archaeological perspective, the autoptic distinction into groups also show a high degree of homogenisation, with a majority group of 15 out of 30 samples (Gr. 1), most of which are included within the largest group of samples identified by the chemical PCA analysis. The mineralogical data confirms this pattern, with the samples exhibiting strong compositional homogeneity featuring mineral phases consistent with the local geology. Notably, some interesting correlation between archaeological provenience, and scientific data can be identified. On a synchronic level, specific associations between the use of particular clays and individual architectural phases identified stratigraphically can be made for

the walls of phase AP 2b (Area B, Late Ubaid period; samples nos. 61, 62, and 63) and the 3rd millennium BCE wall W.458 (Area E, ED III/early Akkadian period; samples nos. 79, 80, and earthen plaster sample no. 302/9). The three samples from phase AP 2b all belong to the autoptic Gr. 4 and show a clear correlation in terms of colour measurements – samples all fall between a* (mean) values of 3–4, and b* (mean) values of 13–16, with chemical PCA data showing differentiation of these samples from the majority group based on higher concentration of CaO. On the other hand, samples nos. 79, 80, and 302/9 all belong to Gr. 6 and, mineralogically, show an absence of amphiboles, serpentine, palygorskite, and pyroxenes. No other clear correspondences appear between the autoptic archaeological groups, the colour measurements and the chemical and mineralogical data.

The homogeneity visible from the mineralogical and chemical data is not confirmed by the petrographic analysis, which reveals diverse preparations based on varying proportions of clay matrix and aggregate content. At least three sub-groups can be distinguished: samples nos. 43, 50, 52, 68, and 79 (including earthen plaster sample no. 302/9) are characterised by a clay-rich matrix with high porosity, attributed to the use of plant fibres as binders and to enhance drying capacity during mudbrick production; sample no. 54 exhibits a distinct division between a zone containing abundant inert materials and a zone with a high clay-rich matrix; samples nos. 62, and 64 present an inverse situation with a composition rich in mineral inert inclusions and low in clay binder.

Differences in the petrographic features of samples correspond to different responses obtained in the hydric and mechanical tests. Mechanical tests have shown that most of the samples are relatively weak in terms of both surface strength and cohesiveness, with mean hardness values ranging from 181.8 HL (sample no. 79) to 300.9 HL (sample no. 51), and with the majority of the samples (20 out of 30) presenting a material loss comprised between 2–6 mg/cm^2 in terms of surface cohesion.

Samples used for the rain resistance test were selected from those with intermediate values in terms of surface hardness and cohesion (see Fig. 10), with the exception of sample no. 79, which is the weakest

in terms of surface hardness. The reason is to test the effects of water on those bricks which, in terms of surface hardness and cohesion, appear to be the weakest, so as to subsequently trial potential practical solutions which, if effective on these, may also prove successful on bricks that are originally more resistant.

Notably, sample no. 79 has resulted to be not only the weakest in terms of surface hardness, but also the least resistant to water; however, the reason does not necessarily lie in its surface hardness, but rather in its clay-rich composition. Indeed, rain resistance test has also demonstrated that the sample no. 62, which exhibits the best water resistance, is not so good in terms of surface cohesion and strength, but it has the most unbalanced matrix-to-aggregate ratio (with high aggregate content), as opposed to samples with a rich clay matrix that decayed more rapidly.

The results indicate that the weakest samples, composed of a clay-rich matrix (e.g., samples nos. 50, 52, and 79), are most susceptible to weathering. In contrast, mudbricks with an unbalanced matrix-to-aggregate ratio favouring high aggregate content (sample no. 62) exhibit superior water resistance.

6 Conclusions

This research has significantly enhanced our understanding of earthen construction techniques at the site of Tell Zurghul/Nigin in southern Mesopotamia.

The results highlight the utilisation of local raw materials over a diachronic period spanning the 5th to the 3rd millennium BCE. Mudbrick samples from the 5th and 4th millennium BCE display a greater variety of mineralogical phases compared to those from the 3rd millennium BCE, suggesting differences in clay sourcing or purification processes during these prehistoric periods which may be further investigated in the future from a chaîne opératoire perspective.

A second objective was to investigate the chemical-mineralogical features and hydro-mechanical properties of these mudbricks to understand their resistance and durability against weathering, informing

conservation strategies at the site. The tests revealed weaknesses and hydrophilicity in the mudbricks, factors that should be considered when planning specific conservation strategies. Despite similarities in mineralogical and chemical characteristics, differences in textural features resulted in varying cohesion and strength properties.

The results indicate that the weakest samples, composed of a clay-rich matrix are most susceptible to weathering, in contrast to mudbricks with an unbalanced matrix-to-aggregate ratio favouring high aggregate content, which exhibit superior water resistance. This drainage capacity could inform ecological conservation solutions, potentially enhancing the durability of earthen archaeological architecture through the production of replica mudbricks with optimised clay-silt-sand proportions.

Funding

The CIVIS3i-MSCA research project EnEAp – Endangered Earthen Architecture project. New Challenges in the Conservation and Restoration Practices of Archaeological Earthen Masonries in Western Asia (2022-EnEAp-231; PI: Luca Volpi) has received funding from the European Union's Horizon 2020 research and innovation programme under grant agreement N°101034324.

This research was also funded by the MICIU/AEI/10.13039/501100011033 and FEDER, UE, under the project PID2023-146405OB-100 (2024-2027).

CRediT authorship contribution statement

Luca Volpi: Conceptualization, Data curation, Formal analysis, Funding acquisition, Investigation, Methodology, Resources, Visualization, Writing – original draft, Writing – review and editing.

Francesco Santoro De Vico: Formal analysis, Investigation, Methodology, Software, Visualization, Writing – original draft, Writing – review and editing.

Anna Arizzi: Conceptualization, Funding acquisition, Methodology, Resources, Software, Supervision, Validation, Writing – review and editing.

Nicola Lanzaro: Investigation, Methodology, Software, Writing – original draft.

Davide Nadali: Resources, Validation, Writing – review and editing.

Acknowledgements

We are grateful to the Iraqi SBAH (State Board of Antiquities and Heritage) of Baghdad for permission to conduct scientific analysis on the archaeological materials.

Supporting information captions

S1 File. Temperature (T), humidity (RH), and rainfall data from reference years (2021, 2011, and 2001). Supplementary information in relation to climatic data for the Tell Zurghul/Nigin area from WorldClim database at https://worldclim.org/.

S2 File. Experimental water. Monthly mean calculation of rain (mm) for reference years (2021, 2011, 2001) and experimental rain (mL) to be applied to the rain resistance test.

S3 File. Python 3.8+ script.

S4 Figure. Polarised optical microscopy (POM) images of all analysed samples (top and bottom) under plane-polarized light (//) and cross-polarized light (┴).

5 File. Spectroscopy values (L*, a*, and b*) for all analysed samples.

S6 File. Surface Hardness test values for all analysed samples.

S7 File. Imbibition time (s) performed through video analysis and first contact angle (CA) measured by the sessile drop method.

References

[1] Cooke L. Conservation approaches to earthen architecture in archaeological contexts. Oxford: BAR; 2010.

[2] Matero F. Mud brick metaphysics and the preservation of earthen ruins. Conserv Manag Archaeol Sites. 2015; 17(3): 209-223. doi:10.1080/13505033.2015.1129798

[3] Agnew N, Taylor M, Balderrama AA, Houben H. 6th International Conference on the Conservation of Earthen Architecture: Las Cruces, New Mexico, USA, October 14–19, 1990: Adobe 90 Preprints. Los Angeles: The Getty Conservation Institute; 1990.

[4] Barnard H, Wendrich WZ, Winkels A, Bos JEMF, Simpson BL, Cappers RTJ. The preservation of exposed mudbrick architecture in Karanis (Kom Aushim), Egypt. J Field Archaeol. 2016; 41(1): 84–100.

[5] Rainer L, Rivera AB, Gandreau D, editors. Terra 2008: Proceedings of the 10th International Conference on the Study and Conservation of Earthen Architectural Heritage, Bamako, Mali, February 1–5, 2008. Los Angeles: The Getty Conservation Institute; 2011.

[6] Volpi L. Western and Central Asia. Satellite Imagery for the Study of Conservation and Restoration of Earthen Architecture. Research Carried Out between July and September 2022. eDAI-F. 2023; 2: 1–21. Doi: 10.34780/cp19-f480

[7] Buccellati G. Conservation qua archaeology at Tell Mozan/Urkesh. In: Agnew N, Bridgland J, editors. Of the Past, for the Future: Integrating Archaeology and Conservation. Proceedings of the Conservation Theme at the 5th World Archaeological Congress, Washington, D.C., 22–26 June 2003. Los Angeles: The Getty Conservation Institute; 2006. p. 73–84.

[8] Kühne H. Zur Erhalt assyrischer Lehmbauten in Tell Schech Hamad, Syrien 2008-2010. Fundiert. Beschützen. bewahren. beachten. 2017; 63–69.

[9] Cooke L. To fill or not to fill? Retrospective backfilling at Merv, Turkmenistan. In: International conference on the Study and Conservation of Earthen Architecture, Tehran, Iran, Preprints of papers: 9th international conference on the Study and Conservation of Earthen Architecture: Terra 2003. Tehran: Deputy of Presentation, Iranian Cultural Heritage Organization; 2003. p. 102–109.

[10] Özdoğan M, Eres Z, Özdoğan E, Demirtaş Yılmaz Ö, Türkoğlu İ, Dereli S, *et al.*. Kırklareli, Asağı Pınar and Kanlıgeçit. Open-Air Site Museum Projects. Istanbul: Kırklareli Kültür Varlıkları Derneği; 2008.

[11] Özdoğan E. Kanlıgeçit – An Anatolian model of an urban center in Eastern Thrace: An overview. In: Pernicka E, Ünlüsoy S, Blum SWE, editors. Early Bronze Age Troy: Chronology, Cultural Development and Interregional Contacts. Proceedings of an International Conference Held at the University of Tübingen May 8–10, 2009. Studia Troica Monographien 8. Bonn: Verlag Dr. Rudolf Habelt GmbH; 2016. p. 197–208.

[12] van Ess M, Blachek JA, Ziegert C. Denkmalschutz des irakischen archäologischen Architekturerbes: aktuelle Erhaltungsprojekte in Uruk (Südirak). In: Dachverband Lehm e.V., editor. LEHM 2020 – Tagungsbeiträge der 8. Internationalen Fachtagung für Lehmbau. Weimar: Eigenverlag Dachverband Lehm; 2020. p. 1–8.

[13] Atalay S, Çamurcuoğlu Cleere D, Hodder S, Moser S, Orbaşli A, Pye E. Protecting and exhibiting Çatalhöyük / Çatalhöyük'ün Korunmasi ve Sergilenmesi. TÜBA-KED. 2010; 8: 7–18.

[14] Balossi Restelli F. A protective shelter at the archaeological site of Arslantepe – Malatya. In: Ahunbay Z, İzmirligil Ü, editors. Management and Preservation of Archaeological Sites. 4th Bilateral Meeting of ICOMOS TURKEY – ICOMOS GREECE, 29 April – 2 May 2002, SİDE (Antalya-Turkey). Istanbul: Side Foundation for Education Culture and Art; 2002. p. 45–49.

[15] Bendakir M, Margueron JC, Vitoux F. Erhaltungsmaßnahmen für die Lehmziegelarchitektur. Bericht über den Stand der Forschungen in Mari. Baghdader Mitt. 2003; 34: 165–179.

[16] Bendakir M. Les vestiges de Mari: La préservation d'une architecture millénaire. Paris: Ed. de la Villette; 2009.

[17] Buccellati G, Bonetti S. Conservation at the core of archaeological strategy. The case of ancient Urkesh at Tell Mozan. Getty Conserv Inst Newsl. 2003; 18(1) :18–21.

[18] Cohen O. Conservation and restoration at Hazor. Near East Archaeol. 2013; 76(2): 118–122. doi:10.5615/neareastarch.76.2.0118

[19] Franco G. Paths to the past: Designing equipment for sustainable management and a culturally aware fruition of the site. In: Marchetti N, Franco G, Musso SF, Spadolini MB, editors. An Integrated Approach for an Archaeological and Environmental Park in South-Eastern Turkey. Tilmen Höyük. Cham: Springer; 2020. p. 129–176.

[20] Frangipane M, Mangano D. The exhibition of a mud-brick monumental complex in a stratified mound: The case of 4th millennium Arslantepe (Malatya) / Bir Höyükte Anitsal Kerpiç Yapi Topluluğunun Sergilenmesi: 4. Binyil Arslantepe (Malatya) Örneği. TÜBA-KED. 2010; 8: 201-214.

[21] Frangipane M. Archeologia, salvaguardia e inclusione ad Arslantepe (Turchia): Il coinvolgimento della comunità locale. Boll Archeol On Line. 2022; 13(1): 19–47.

[22] Hlal F, Pfälzner P. Die Restaurierung des Königspalastes von Qaṭna. In: Pfälzner P, Schmid J, editors. Der Königspalast von Qaṭna Teil 1: Chronologie, Grundriss, Baugeschichte und Bautechniken. Wiesbaden: Harrassowitz Verlag; 2019. p. 311–28.

[23] Dehkordi MH, Vatandoust R, Madjidzadeh Y, Kashi MG. The archaeological site of Konar Sandal, Jiroft, Iran: Conservation of earthen architecture. In: Rainer L, Rivera AB, Gandreau D, editors. Terra 2008: Proceedings of the 10th International Conference on the Study and Conservation of Earthen Architectural Heritage, Bamako, Mali, February 1–5, 2008. Los Angeles: The Getty Conservation Institute; 2011. p. 183–188.

[24] Diab M. Conservation of mud-brick structures at Tell es-Sultan. In: Nigro L, Taha H, editors. Tell es-Sultan/Jericho in the context of the Jordan Valley. Site management, conservation and sustainable development. Rome: «La Sapienza» Expedition to Palestine & Jordan; 2006. p. 227–236.

[25] Gandreau D, Sadozaï C, Moriset S. Earthen architecture on archaeological sites: Sustainability principles vs decay processes. In: Daneels A, Torras Freixa M, editors. Earthen Construction Technology. Proceedings of the XVIII UISPP World Congress (4-9 June 2018, Paris, France). Oxford: Archaeopress; 2021. p. 5–14.

[26] Gelin M. Treatment, conservation and restoration of mudbrick structures. In: Nehmé L, al-Talhi D, Villeneuve F, editors. Hegra 1. Report on the first excavation season at Madâ'in Sâlih-2008 Saudi Arabia. Riyadh: Saudi Commission for Tourism and Antiquities; 2010. p. 28–46.

[27] Nigro L, Taha H. Renewed excavations and restorations at Tell es-Sultan/Ancient Jericho. Fifth season – March-April 2009. Sci Antichità. 2009; 15: 731–744.

[28] Nigro L, Ripepi G, Hamdan I, Yasine J. The Jericho Oasis Archaeological Park - 2015 Interim Report Italian-Palestinian Cooperation for Protection and Valorization of Archaeological Heritage. Vicino Oriente. 2015; 19: 215–243.

[29] Sadozaï C. Préservation de l'architecture de terre en Asie centrale. L'exemple du site protohistorique d'Ulug dépé (Turkménistan). Cah Asie Cent. 2013; 21/22: 623–630.

[30] Sadozaï C, Gandreau D. Outils stratégiques et techniques pour la conservation des sites archéologiques en terre. In: Baud A, Charpentier G, editors. Chantiers et matériaux de construction de l'antiquité à la révolution industrielle en orient et en occident. Actes du colloque tenu au château de Guédelon (23-25 septembre 2015). Lyon: Maison de l'Orient et de la Méditerranée – Jean Pouilloux; 2020. p. 255–269.

[31] Seeher J. A mudbrick city wall at Hattusa: Diary of a reconstruction. Istanbul: Ege Yayinlari; 2007.

[32] Ebrahimi A. Researches on mud-brick and straw-clay in "Choga Zanbil" World Heritage Site. In: International conference on the Study and Conservation of Earthen Architecture, Tehran, Iran, Preprints of papers: 9th international conference on the Study and Conservation of Earthen Architecture: Terra 2003. Tehran: Deputy of Presentation, Iranian Cultural Heritage Organization; 2003. p. 133–140.

[33] Marchetti N. Never ending story. Responsibility, strategy and sustainability in managing an archaeological excavation and its public presentation from its inception to beyond its termination. In: Marchetti N, Franco G, Musso SF, Spadolini MB, editors. An Integrated Approach for an Archaeological and Environmental Park in South-Eastern Turkey. Tilmen Höyük. Cham: Springer; 2020. p. 11–42.

[34] Mari C, Capriani U, Finotelli F, Marabini S. Restauro e valorizzazione turistica del sito di Ebla (Siria). Prime fasi sperimentali di restauro, consolidamento e protezione. In: Matthiae P, Enea A, Peyronel L, Pinnock F, editors. Proceedings of the First International Congress on the Archaeology of the Ancient Near East. Rome: Scienze e Lettere; 2000. p. 929–944.

[35] Nadali D. How to deal with adobe architecture in the Ancient Near East: The case of Ebla in Syria. Heritage. 2023; 6(2) :1856–1870. doi:10.3390/heritage6020099

[36] Pigott V, Butterbaugh DJ. A programme in experimental mudbrick preservation at Hasanlu Tepe. Iran. 1978; 16: 161–167.

[37] Pinnock F. The archaeological park of Ebla. A long-term plan for the site and its region. In: Abdul Massih J, Nishiyama S, editors. Archaeological Explorations in Syria 2000-2011. Proceedings of ISCACH-Beirut 2015. Oxford: Archaeopress; 2018. p. 297–307.

[38] Fragnoli P, Cereda S, Liberotti G. Earthen stories: Cross-craft strategies in raw material procurement and production at the tell site of Arslantepe (Türkiye) during the 4th millennium BCE. J Archaeol Sci Rep. 2024; 54: 104447. doi:10.1016/j.jasrep.2024.104447

[39] Liberotti G, Rovero L, Stipo G, Tonietti U. Mechanical investigation on adobe samples belonging to the archaeological site of Arslantepe (Malatya, Turkey). J Mater Environ Sci. 2016; 7(10): 3656–3666.

[40] Lorenzon M, Cutillas-Victoria B, Holmqvist E, Gkouma M, Vrydaghs L, Lichtenberger A, Schreiber T, Zardaryan M. Exploring mudbrick architecture and its re-use in Artaxata, Armenia during the 1st millennium BC: A multidisciplinary study of earthen architecture in the Armenian Highlands. PLoS ONE. 2023; 18(10): e0292361. doi:10.1371/journal.pone.0292361

[41] Lorenzon M, Cutillas-Victoria B, Itkin E, Fantalkin A. Masters of mudbrick: Geoarchaeological analysis of Iron Age earthen public buildings at Ashdod-Yam (Israel). Geoarchaeology. 2024; 39(1): 35–62. doi:10.1002/gea.21977

[42] Lorenzon M, Cutillas-Victoria B, Lichtenberger A, Tal O. Of mudbrick and stone: A geoarchaeological view on innovations in building practices at Hellenistic Tell Iẓṭabba. J Archaeol Sci Rep. 2024; 54: 104389. doi:10.1016/j.jasrep.2024.104389

[43] Love S. The geoarchaeology of mudbricks in architecture: Methodological study from Çatalhöyük, Turkey. Geoarchaeology. 2012; 27: 140-156. doi:10.1002/gea.21401

[44] Love S. Field methods for the analysis of mud brick architecture. J Field Archaeol. 2017; 42: 351–363. doi:10.1080/00934690.2017.1345222

[45] Setti M, Arizzi A, Nieto P, Velilla Sánchez N, Cultrone G, d'Alfonso L. From ancient construction, through survival, towards modern conservation: Characterization of fine-grained building material at Niğde-Kınık Höyük (Cappadocia, Turkey). Archaeol Anthropol Sci. 2021; 13: 79. doi:10.1007/s12520-021-01309-0

[46] Nadali D. Area D: Stratigraphy and architecture of the "Mountain Lifted Above all (Other) Houses" at Tell Zurghul, Ancient Nigin, Iraq. In: Nadali D, Polcaro A, editors. The Italian

Archaeological Excavations at Tell Zurghul, Ancient Nigin, Iraq. Final Report of the Seasons 2015–2017. Quaderni di Vicino Oriente XVI. Rome: Sapienza Università di Roma; 2020. p. 35–52.

[47] Nadali D, Verderame L. Fragments of the third millennium BC from Nigin. Iraq. 2021; 83: 105–118.

[48] Nadali D, Volpi L. The 3rd millennium BCE pottery sequence of southern Mesopotamia: Pottery chronology as seen from Tell Zurghul/Nigin, Mound A, Iraq. Iraq. 2025; 1–39. doi:10.1017/irq.2024.13

[49] Volpi L. An assessment on the Ubaid pottery from Area B, Tell Zurghul (Southern Iraq). In: Nadali D, Polcaro A, editors. The Italian Archaeological Expedition at Tell Zurghul, Ancient Nigin, Iraq. Final Report of the Seasons 2015–2017. Rome: Sapienza Università di Roma (Quaderni di Vicino Oriente XVI); 2020. p. 53–87.

[50] Volpi L. Revisiting the South: a typochronological approach to the analysis of the Ubaid pottery based on the new data from Tell Zurghul (Dhi Qar, Iraq). Paléorient. 2022; 48(1): 175–199. doi:10.4000/paleorient.1765

[51] Goodman R. Tides of change: A geoarchaeological study of city and state formation in Lower Mesopotamia [dissertation]. Philadelphia: University of Pennsylvania; 2023.

[52] Sissakian VK, Fouad SFA. The origin of Amadiya plateau, Dohuk governorate, north Iraq. Iraqi Bull Geol Min. 2011; 7(1): 93–98.

[53] Sissakian VK, Fouad SFA. Geological map of Iraq, scale 1:1000 000, 4th edition, 2012. Iraqi Bull Geol Min. 2015; 11(1) :9–16.

[54] Aqrawi AA, Evans G. Sedimentation in the lakes and marshes (Ahwar) of the Tigris-Euphrates Delta, southern Mesopotamia. Sedimentology. 1994; 41(4): 755–776.

[55] Jassim SZ, Goff JC, editors. Geology of Iraq. Prague; Brno: Dolin; Moravian Museum; 2006.

[56] Rubel F, Kottek M. Observed and projected climate shifts 1901–2100 depicted by world maps of the Köppen-Geiger climate classification. Meteorol Z. 2010; 19(2): 135–141.

[57] Nadali D, Polcaro A. Excavating the ancient state of Lagash. The new evidence from Tell Zurghul/Nigin. In: Nadali D, Pittman H, Polcaro A, editors. Ancient Lagash. Current Research and Future Trajectories. Proceedings of the Workshop held at the 10th ICAANE in Vienna, April 2016. Vienna: Austrian Academy of Sciences Press; 2022. p. 15–30.
[58] Polcaro A. The excavations in Area A: The discovery of a multifunctional building of the 4th–3rd millennium BC. In: Nadali D, Polcaro A, editors. The Italian Archaeological Expedition at Tell Zurghul, Ancient Nigin, Iraq. Final Report of the Seasons 2015–2017. Quaderni di Vicino Oriente XVI. Rome: Sapienza Università di Roma; 2020. p. 19–34.
[59] Quinn PS. Thin section petrography, geochemistry & scanning electron microscopy of archaeological ceramics. Oxford: Archaeopress; 2022.
[60] Charola AE, Otero J, Depriest PT, Koestler RJ, editors. Built Heritage Evaluation. Manual Using Simple Test Methods. Washington, D.C.: Smithsonian Institution Scholarly Press; 2021.
[61] Krainer S, Hirn U. Contact angle measurement on porous substrates: Effect of liquid absorption and drop size. Colloids Surf A Physicochem Eng Asp. 2021; 619: 126503. doi:10.1016/j.colsurfa.2021.126503
[62] Arizzi A, Viles H, Martín-Sanchez I, Cultrone G. Predicting the long-term durability of hemp–lime renders in inland and coastal areas using Mediterranean, tropical and semi-arid climatic simulations. Sci Total Environ. 2016; 542: 757–770. doi:10.1016/j.scitotenv.2015.10.141
[63] Dakheel AA. Drawing curves of the rainfall intensity duration frequency (IDF) and assessment equation intensity rainfall for Nasiriyah City, Iraq. J Thi-Qar Univ. 2017; 12(2): 63–78. doi:10.32792/utq/utj/vol12/2/6
[64] Sissakian VK, Al-Ansari N, Adamo N, Abdullah M, Laue J. Stratigraphy of the Mesopotamian Plain: A critical review. J Earth Sci Geotech Eng. 2020; 10(4): 27–56.

[65] Altaweel M, Watanabe C. Assessing the resilience of irrigation agriculture: Applying a social-ecological model for understanding the mitigation of salinization. J Archaeol Sci. 2012; 39(4): 1160–1171.

[66] Artzy M, Hillel D. A defense of the theory of progressive soil salinization in ancient southern Mesopotamia. Geoarchaeology. 1988; 3(3): 235–238. doi:10.1002/gea.3340030306

[67] Jacobsen T, Adams RM. Salt and silt in ancient Mesopotamian agriculture. Science. 1958; 128(3334): 1251–1258. doi:10.1126/science.128.3334.1251

[68] Jacobsen T. Salinity and irrigation agriculture in antiquity: Diyala Basin archaeological report on essential results, 1957–58. Malibu, CA: Undena Publications; 1982.

[69] Powell MA. Salt, seed and yields in Sumerian agriculture: A critique of the theory of progressive salinization. Z Assyriol. 1985; 75: 7–38.

Fig. 1

Click here to access/download;Figure;Fig. 1.tif

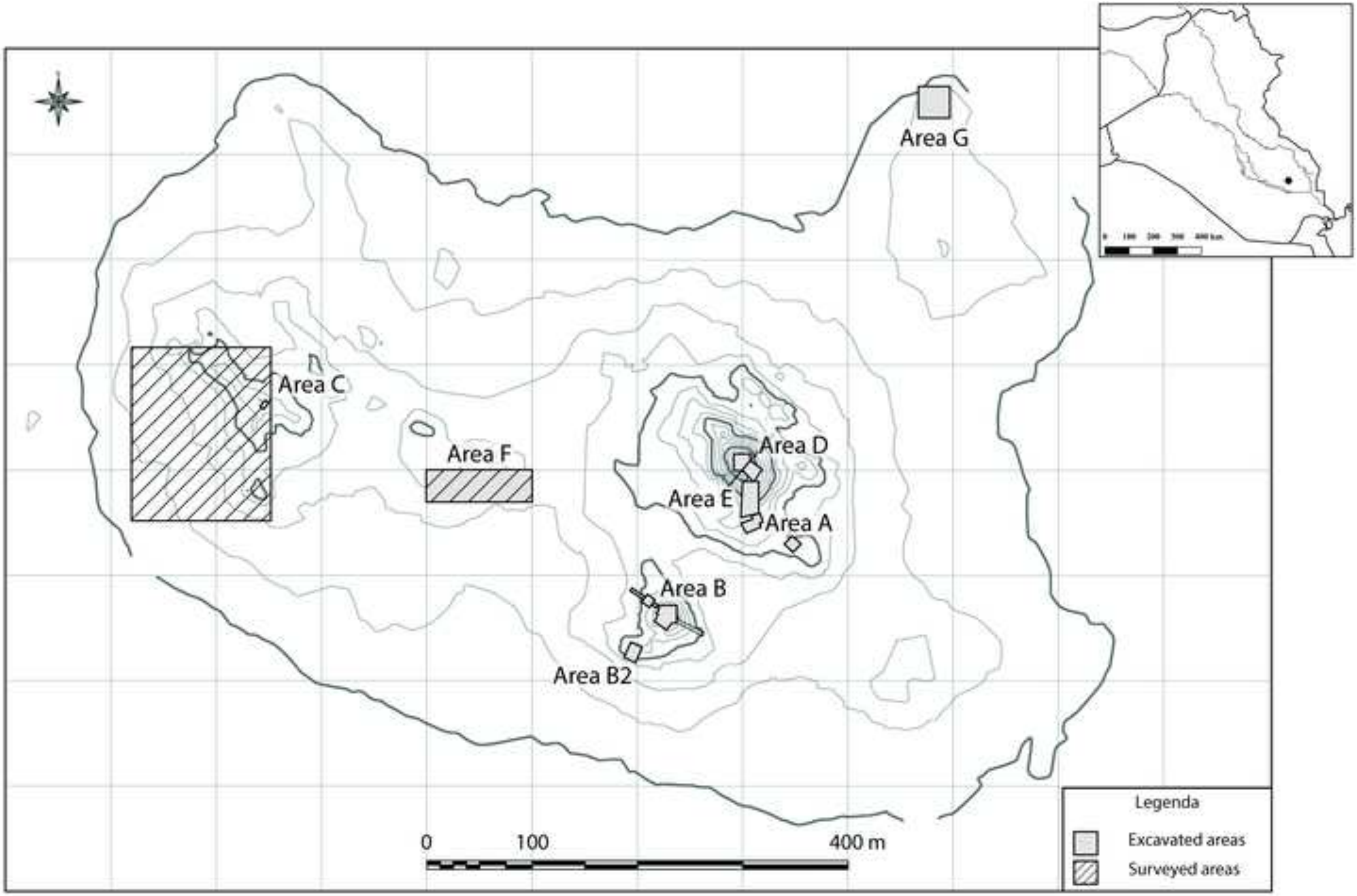

Fig. 2

Click here to access/download;Figure;Fig. 2.tif

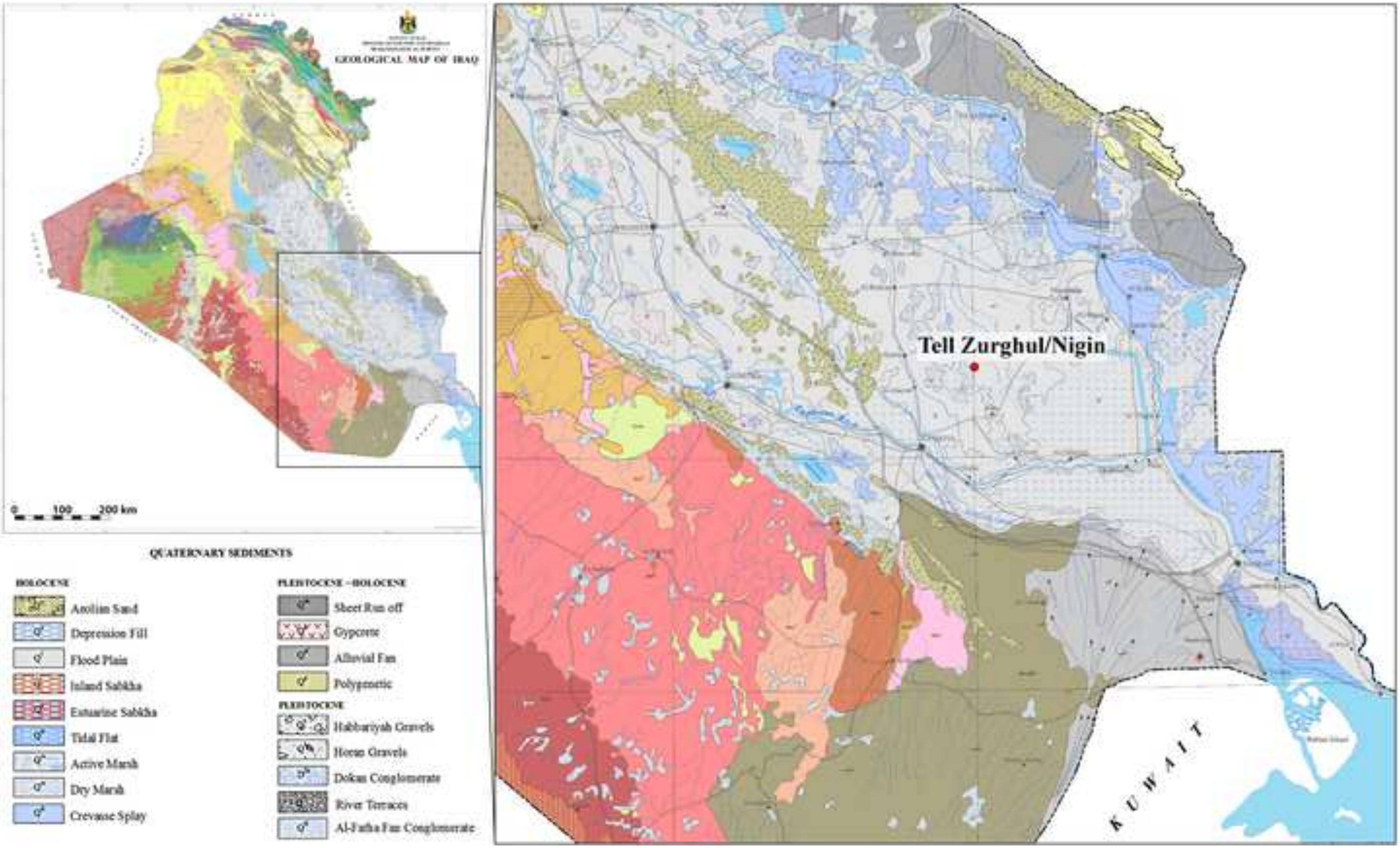

Fig. 3

Click here to access/download;Figure;Fig. 3.tif

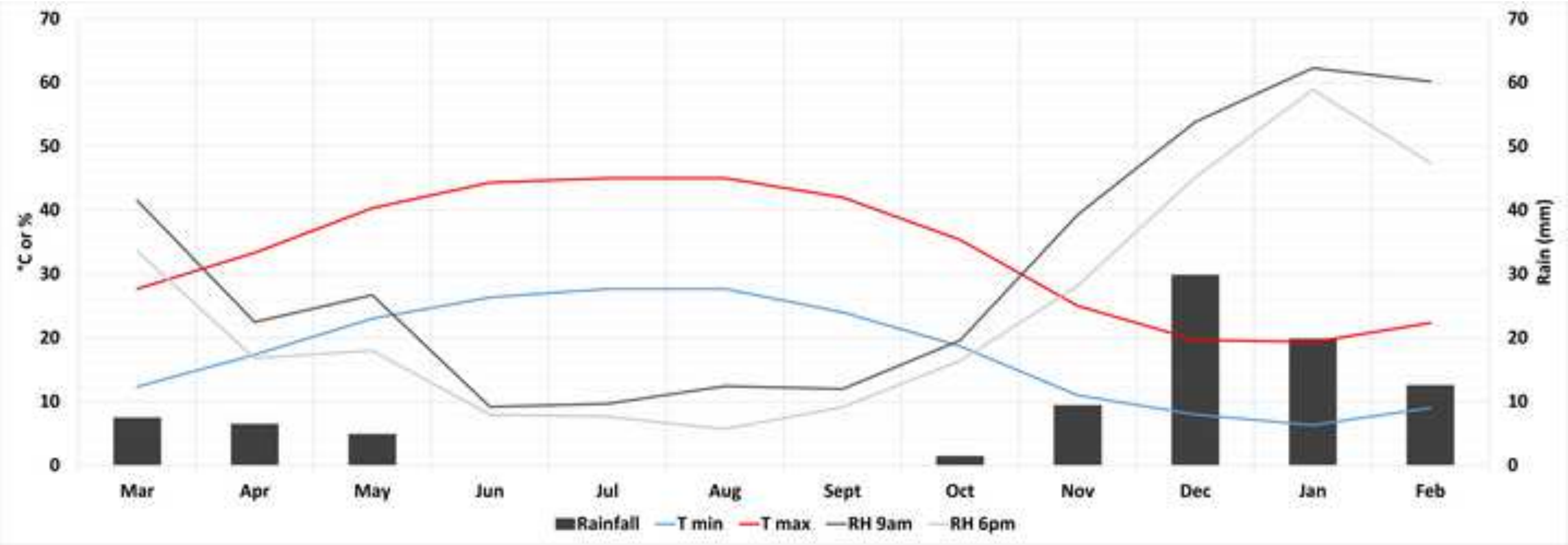

Fig. 4

Click here to access/download;Figure;Fig. 4.tif

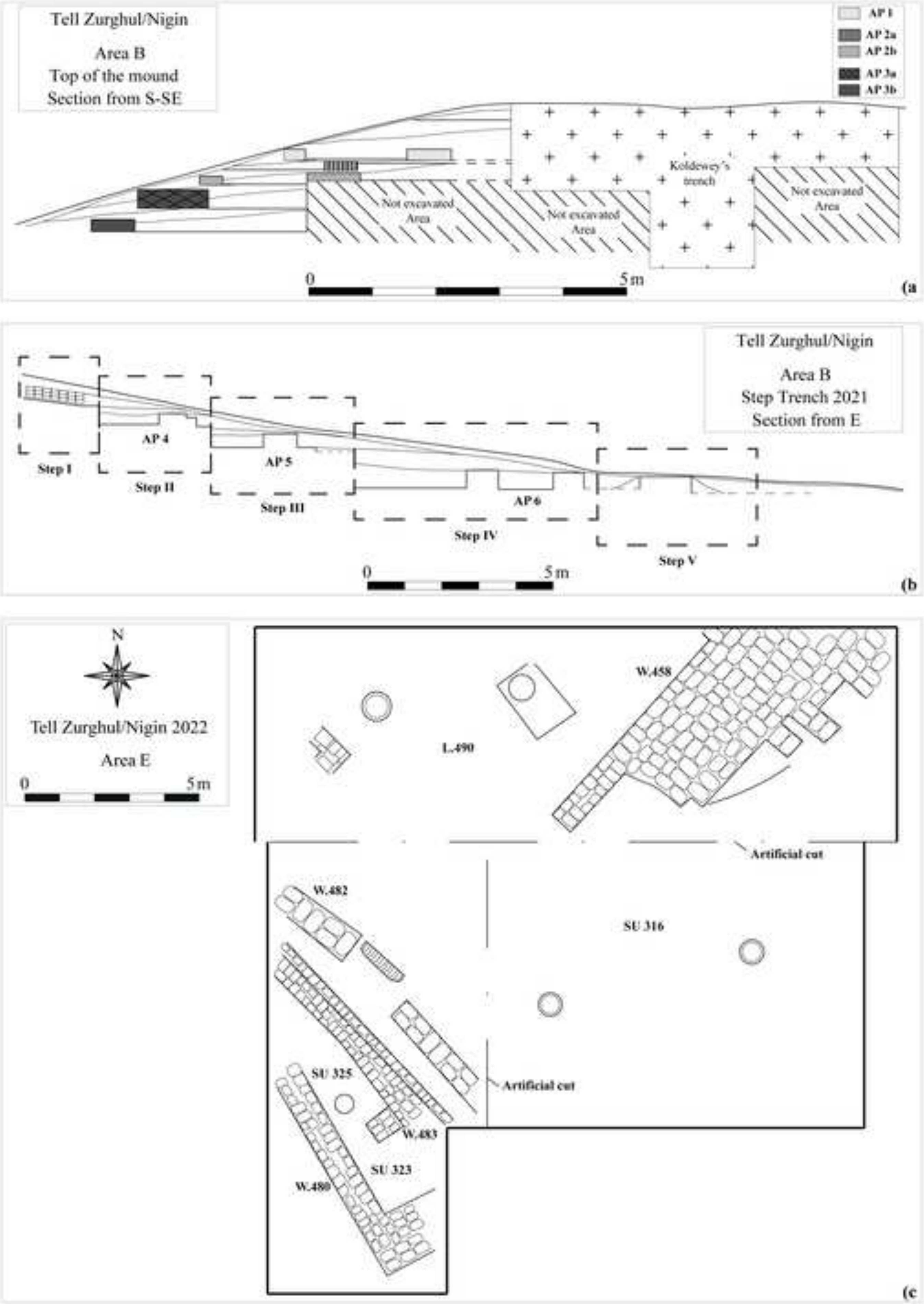

Fig. 5

Click here to access/download;Figure;Fig. 5.tif

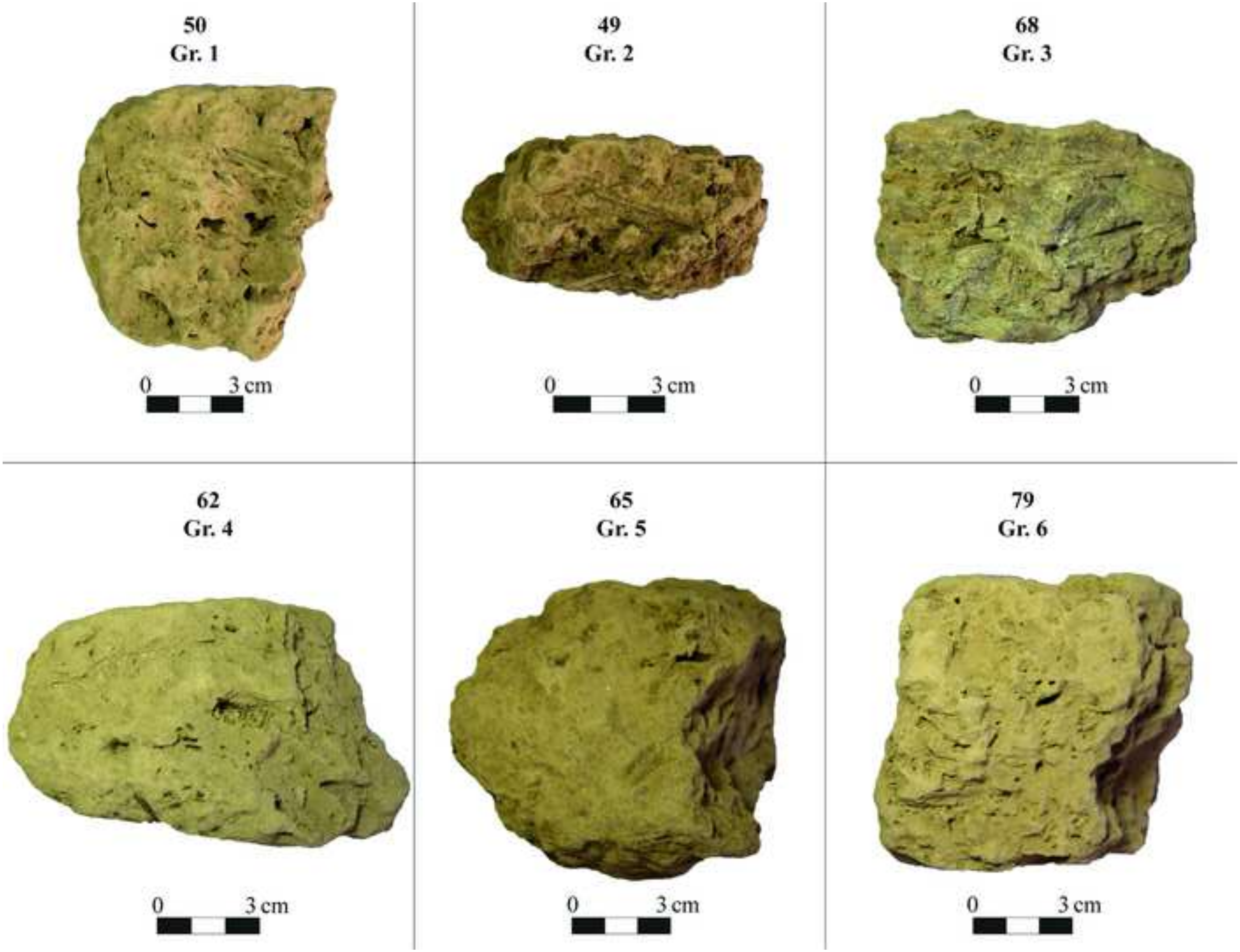

Fig. 6

Click here to access/download;Figure;Fig. 6.tif

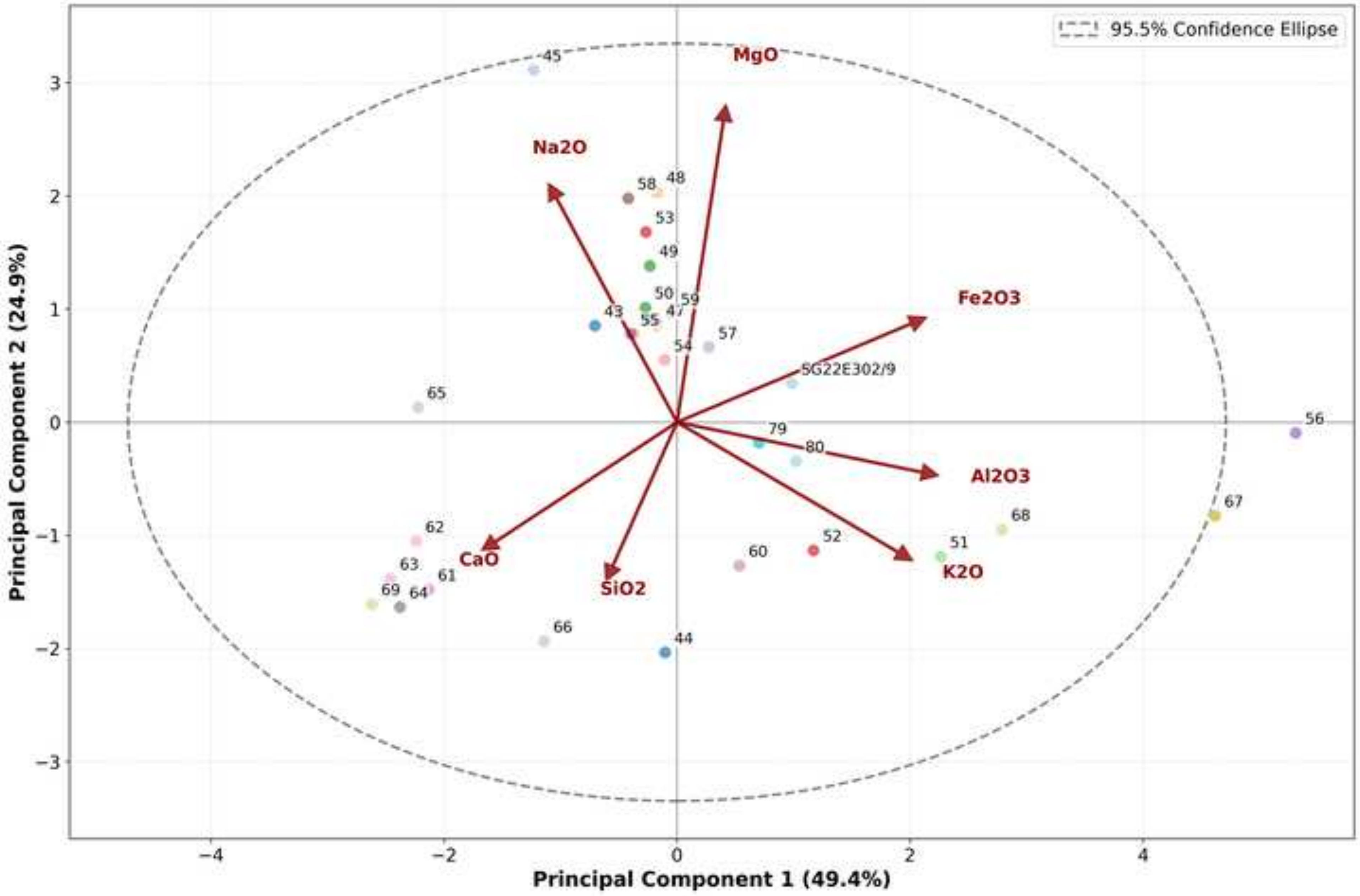

Fig. 7 Click here to access/download;Figure;Fig. 7.tif

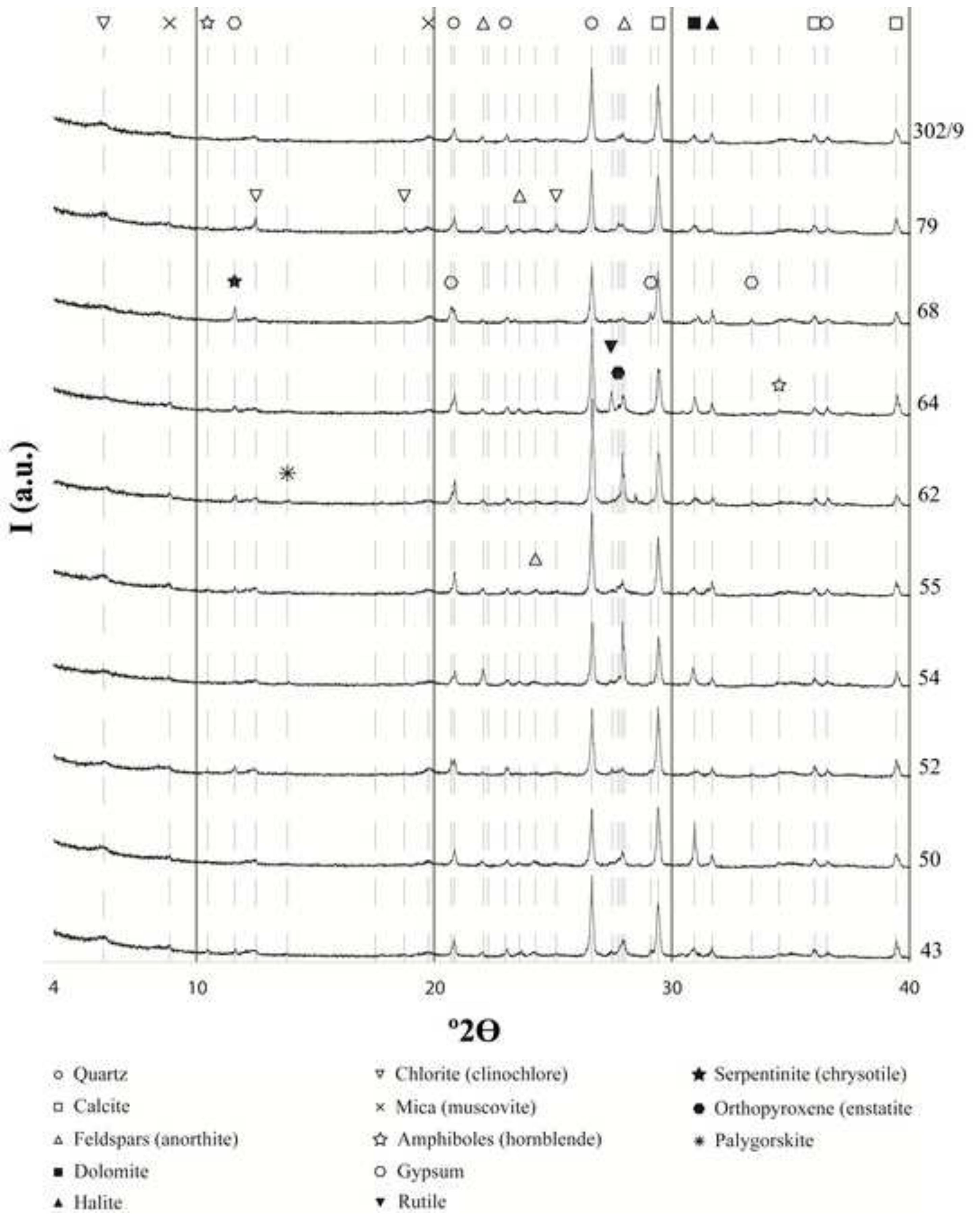

Fig. 8

Click here to access/download;Figure;Fig. 8.tif

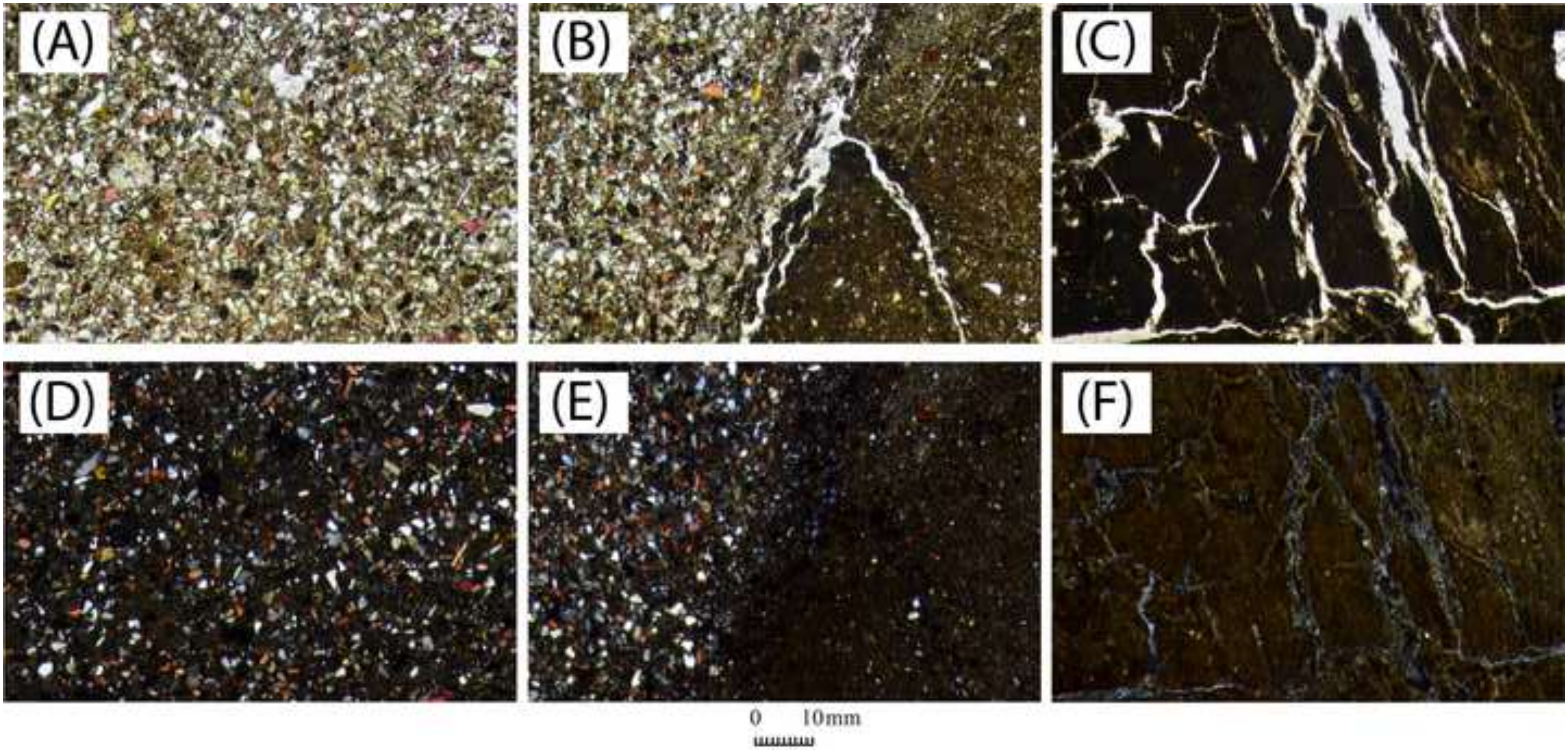

Fig. 9 Click here to access/download;Figure;Fig. 9.tif

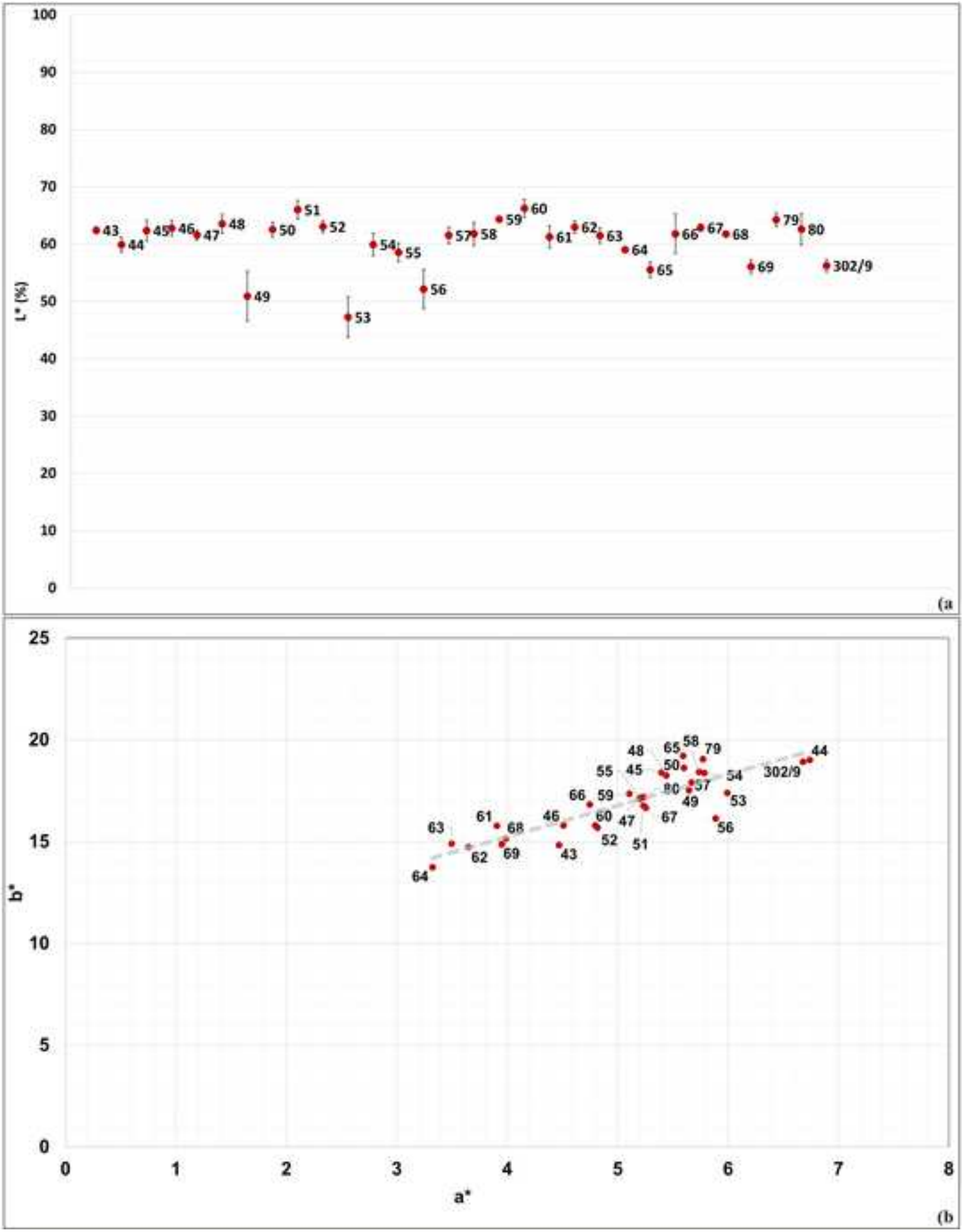

Fig. 10 Click here to access/download;Figure;Fig. 10.tif

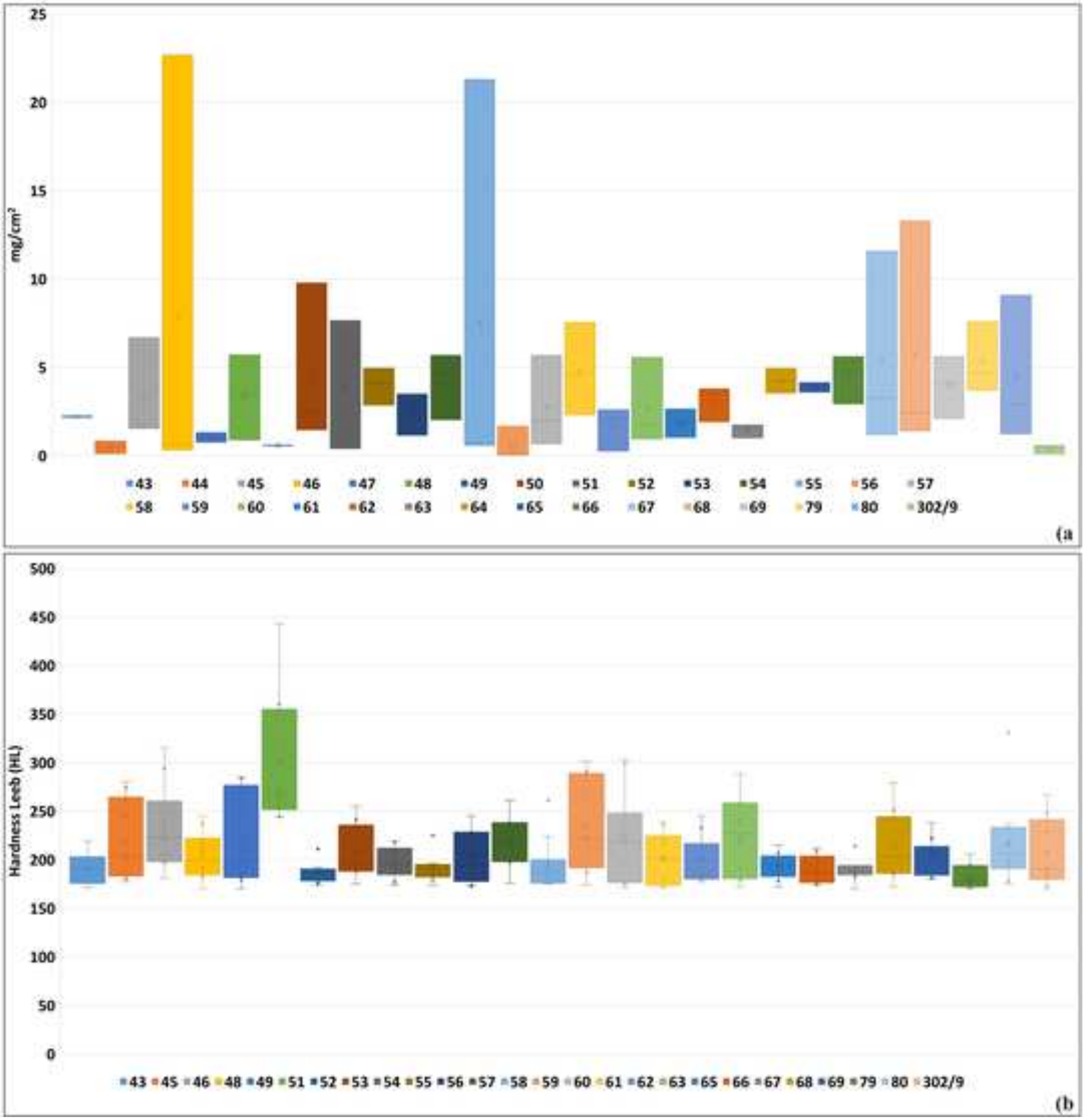

Click here to access/download;Figure;fig. 11.tif

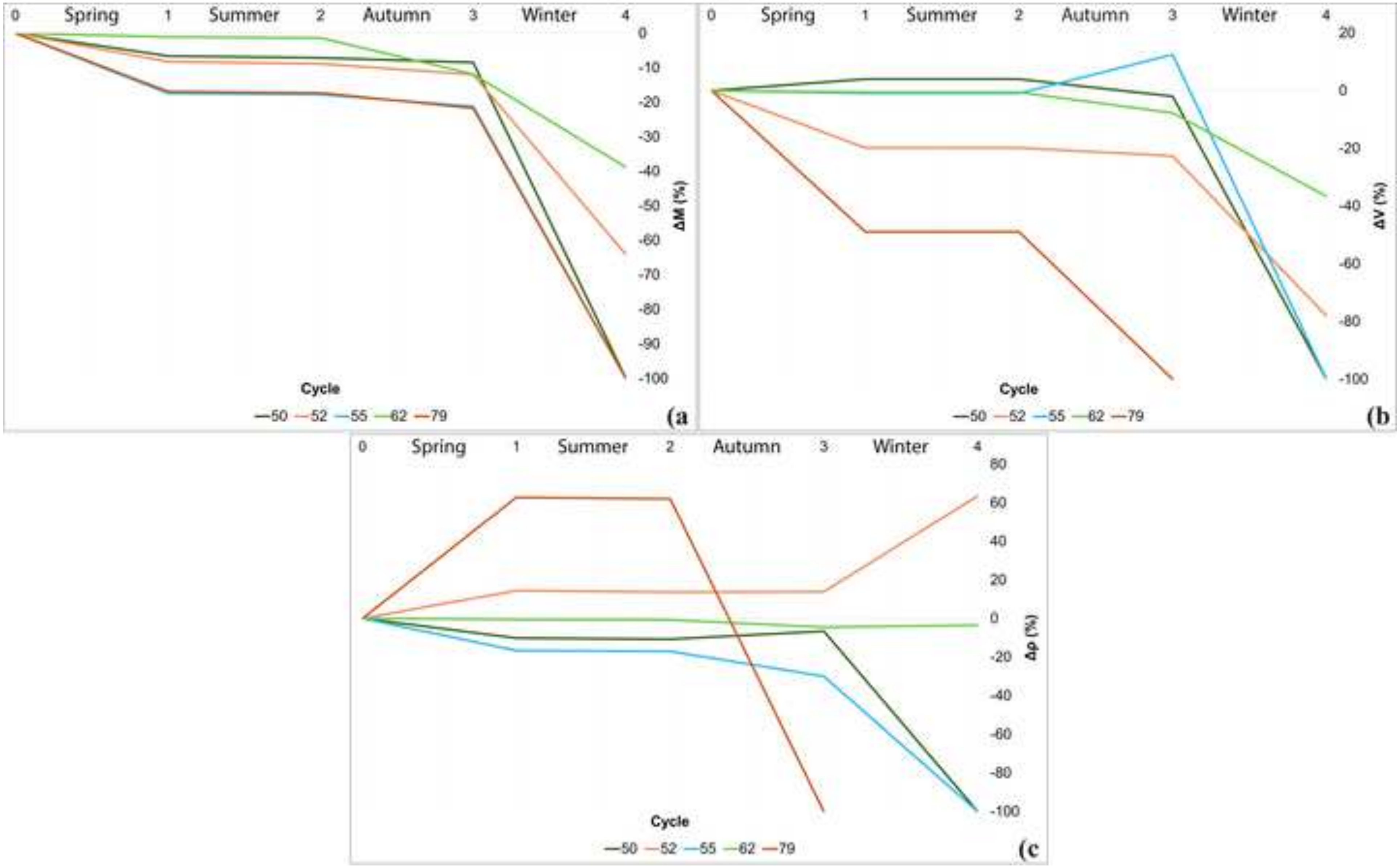

Fig. 12

Click here to access/download;Figure;Fig. 12.tif

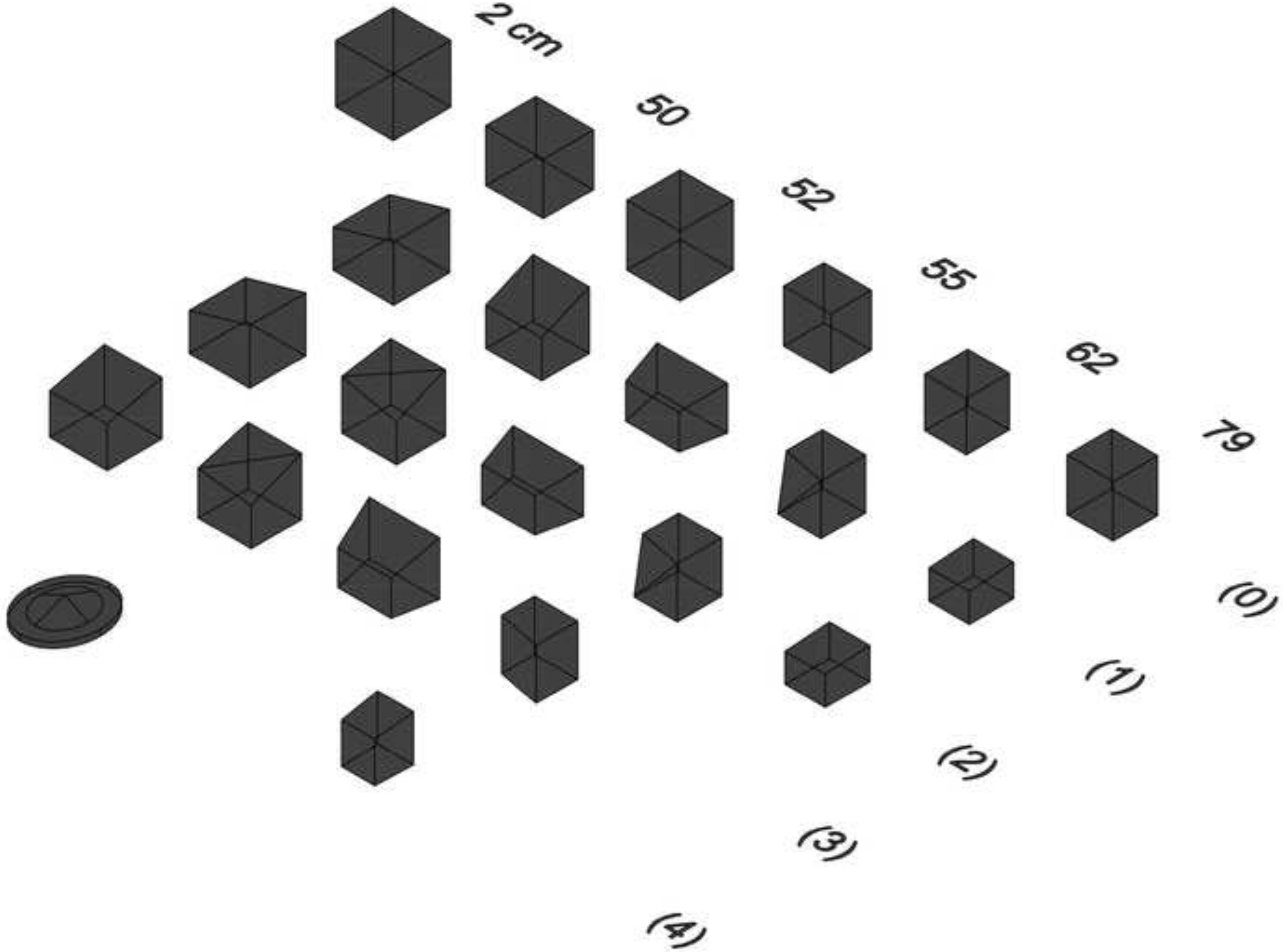

Click here to access/download
Supporting Information - Compressed/ZIP File Archive
Supplementary Materials.zip

Data Review URL

Click here to download Data Review URL
http://doi.org/10.21950/Z7EFXL
