# Supplementary material for: Chemical-mineralogical features and physical properties of archaeological adobe: The evidence from Tell Zurghul/Nigin (Dhi Qar, Iraq)
Source: PLoS One. 2026 Feb 23;21(2):e0342009. doi: 10.1371/journal.pone.0342009 (PMC12928471; doi:10.1371/journal.pone.0342009)
Supplement: Attachment — Submitted filename: Response to Reviewers.docx [file pone.0342009.s008.docx]

To the kindest attention of:

the Academic Editor of *PLOS ONE* Journal,

in the person of Dr. Santanu Banerjee,

Dear Academic Editor Dr. Banerjee,

Thank you very much for your kind assistance and support throughout the review process.

I am writing to resubmit our manuscript entitled *“Chemical-mineralogical features and physical properties of archaeological adobe: Evidence from Tell Zurghul/Nigin (Dhi Qar, Iraq)”* following the revision process for consideration as a PLOS ONE Research Article.

The manuscript has been carefully revised by all authors in accordance with the comments and recommendations provided by the Academic Editor and the two peer reviewers.

Below, we summarise how all editorial and technical requirements have been addressed.

1. The revised manuscript now fully complies with PLOS ONE’s style requirements.
2. The questionnaire on inclusivity in global research has been uploaded as Supporting Information.
3. The information reported in the *Funding Information* and *Financial Disclosure* sections is now fully consistent.
4. The following statement has been added to the manuscript: *“The funders had no role in study design, data collection and analysis, decision to publish, or preparation of the manuscript.”*
5. The minimal dataset necessary to replicate the study has been uploaded to Figshare (10.6084/m9.figshare.30933299).
6. Data availability has been clearly stated, and the data are now publicly accessible.
7. The copyright for Figures 1, 4, 5, and 6 belongs to the *Missione Archeologica Italiana a Nigin*, directed by Prof. Davide Nadali (Sapienza Università di Roma) and field-directed by myself, Luca Volpi (Universidad Autónoma de Madrid). As both copyright holders and authors of the present article, we agree to publish these figures under a CC BY 4.0 licence.

For Figure 1, the image is reprinted from an Open Access journal published under CC BY 4.0 license. For the other images, the requested files have been attached. We kindly ask you to confirm whether the figure captions are appropriate.

8. The copyright for Figure 2 belongs to the *Iraqi Bulletin of Geology and Mining*, issued by the Iraq Geological Survey (GEOSURV, Iraq), Ministry of Industry and Minerals, Baghdad. The journal operates under a Creative Commons Attribution 4.0 International Licence, which permits redistribution and adaptation provided appropriate credit is given. The remixed map used in our article is available at: <https://ibgm-iq.org/ibgm/index.php/ibgm/article/view/263>.

The licensing statement is reported at the bottom of the webpage. On this basis, we understand that Figure 2 may be published under a CC BY 4.0 licence. We would appreciate confirmation that the corresponding figure caption is acceptable.

9. All reviewer comments have been carefully considered and addressed. We sincerely thank the reviewers for their constructive feedback and interest in our work. In particular, we have fully taken on board Reviewer #1’s comments regarding the conservation aspects of the manuscript. As a result, we have decided to retain the paper strictly as a material study of Tell Zurghul/Nigin. References to conservation work have therefore been removed from the title – which could have been misleading – and systematically eliminated throughout the text. A single reference has been retained solely to clarify that the material study was developed within a broader research framework concerned with adobe conservation and is intended as a baseline, reference analysis.

Given this shift in focus, we did not include the additional references suggested by the reviewer, as they were specifically aimed at expanding the conservation discussion, which has been intentionally excluded from this article.

In line with these revisions, we have slightly modified the title of the paper. Furthermore, we have replaced all occurrences of the term *“mudbrick”* with *“adobe”*, for reasons of terminological accuracy, ethical clarity, and inclusivity.

10. The reference list has been thoroughly reviewed and is now complete and accurate.

We hope that the revised manuscript meets the standards of PLOS ONE and we remain at your disposal should any further clarification or modification be required.

Thank you again for your time and consideration.

Yours sincerely,

Luca Volpi
